# Impacts of medication non-adherence to major modifiable stroke-related diseases on stroke prevention and mortality: a meta-analysis

**DOI:** 10.1007/s00415-023-11601-9

**Published:** 2023-02-27

**Authors:** Okti Ratna Mafruhah, Yen-Ming Huang, Hsiang-Wen Lin

**Affiliations:** 1 School of Pharmacy and Graduate Institute, College of Pharmacy, China Medical University, Taichung City, 406040 Taiwan; 2 Department of Pharmacy, Universitas Islam Indonesia, Daerah Istimewa Yogyakarta, 55584 Indonesia; 3 Graduate Institute of Clinical Pharmacy, College of Medicine, National Taiwan University, Taipei City, 100025 Taiwan; 4 School of Pharmacy, College of Medicine, National Taiwan University, Taipei City, 100025 Taiwan; 5 Department of Pharmacy, National Taiwan University Hospital, Taipei City, 100229 Taiwan; 6 Department of Pharmacy, China Medical University Hospital, Taichung City, 404332 Taiwan; 7 Department of Pharmacy System, Outcomes and Policy, College of Pharmacy, University of Illinois at Chicago, Chicago, IL 60612 USA

**Keywords:** Medication adherence, Modifiable diseases, Stroke prevention, Stroke occurrence, Mortality

## Abstract

**Background:**

Medication adherence is one of the crucial attempts in primary stroke prevention. The available evidence lacks comprehensive reviews exploring the association of medication adherence with stroke prevention.

**Objectives:**

To investigate the effects of non-adherence to medications used to treat the modifiable risk of diseases on stroke-associated outcomes in primary stroke prevention.

**Methods:**

Study records were searched from PubMed, Embase, and CINAHL. Those studies reported risks relevant to stroke-associated outcomes and medication non-adherence for patients diagnosed with four modifiable stroke-related diseases (atrial fibrillation [AF], hyperlipidemia, hypertension, and type 2 diabetes mellitus) but without stroke history were included for meta-analysis and further subgroup, sensitivity, and publication bias analyses. A random effect model was performed to analyse the pooled risk estimates of relative risk (RR) and 95% confidence intervals (CIs).

**Results:**

Thirty-nine studies (with 2,117,789 participants in total) designed as cohort or case–control studies were included. Those patients presenting with four stroke-related diseases and categorised as medication non-adherent tended to result in stroke and/or associated death (all pooled RR ≥ 1 and 95% CI did not include 1). The findings of stratification and sensitivity analysis for each stroke-related disease showed a similar trend. Non-adherent patients with AF were prone to stroke occurrence (RR 1.852; 95% CI 1.583–2.166) but inclined to reduced bleeding (RR 0.894; 95% CI 0.803–0.996). The existence of publication bias warrants further interpretation.

**Conclusions:**

Non-adherence to medications for the four stroke-related diseases contributes to the development of stroke and/or mortality in primary stroke prevention. More efforts are needed to improve patients’ medication adherence.

**Supplementary Information:**

The online version contains supplementary material available at 10.1007/s00415-023-11601-9.

## Introduction

Stroke is a neurological disease characterised by reduced or blocked blood flows to the brain or ruptures of blood vessels that cause cerebral haemorrhage or cerebrovascular tissue damage. Based on the report of the Centers for Disease Control and Prevention, stroke is a leading cause of long-term physical impairment and mortality, and one in six deaths of cardiovascular diseases resulted from stroke [1]. Over the last 3 decades, the incidence and prevalence of stroke have grown drastically, with the number of deaths rising by more than 40% worldwide. The estimated cases of stroke will continuously increase because of the suboptimal management of stroke-related diseases (e.g., hypertension, atrial fibrillation [AF], hyperlipidemia, and diabetes) [2] and the newly emerging infectious disease, COVID-19, as a potential combination risk of acute ischemic stroke among patients with chronic diseases [3].

Stroke occurs suddenly and tends to happen or arise among patients with four major modifiable diseases (i.e., hypertension, AF, hyperlipidemia, and diabetes) [4]. Yet, around 40% of patients with stroke admitted to hospitals fail to identify the signs and symptoms of stroke [5]. Treatments for stroke attacks are limited and must be carried out immediately to get patients not only to recover physical functionality but also to reduce risks of death [6]. Thus, the primary prevention of the first stroke event is considered a substantial and the most important step to decreasing stroke episodes [7].

Primary stroke prevention refers to all attempts implemented to avoid the first stroke attack among people without a stroke history [8]. Stroke preventions target controlling major modifiable risks or diseases (i.e., blood pressure, body-mass index, blood glucose, lipid level [2, 7], and AF) [7, 9] through medical treatments, diet adjustment, and lifestyle modification. Several trials have proven that medications for the major stroke-related diseases (e.g., antihypertensive agents [10], statins [11], and antidiabetic agents [12]) and embolism prevention (e.g., non-vitamin K antagonist oral anticoagulants [13]) are effective in reducing major risks of stroke and mortality.

Some studies demonstrated that medication adherence is essential in stroke management to reach optimal clinical outcomes [14]. Previous meta-analyses reported that higher adherence to cardiovascular medications was associated with a decreased risk of coronary heart disease, stroke, and all-cause mortality [15, 16]. Although medication adherence benefits patients at risk of stroke, around forty to fifty percent of patients do not take their cardiovascular medications as prescribed [15, 17]. Thus, concerns about the clinical outcome and the extent of the risk of medication non-adherence still exist. Previous reviews evaluated the effect of non-adherence to anticoagulants on stroke-associated outcomes for patients diagnosed with AF without stroke before [18, 19]. The findings indicated that medication non-adherence was not associated with bleeding risk and stroke events [20]. Other studies targeted medication adherence to individual cardiovascular-related medications (i.e., antihyperlipidemic and antihypertensive medications) and relevant outcomes (e.g., stroke events and all-cause mortality) but did not consider or adjust for the other major disease risk factors (e.g., diabetes mellitus [DM]) [15, 16, 21, 22]. Their findings reported that medication adherence was associated with decreased risk of stroke events and all-cause mortality. The inconsistent findings across different studies raised questions about the exact association linking medication adherence to risks of stroke events. In addition, none of these studies reported the effect of medication non-adherence on the risk of stroke among patients across various modifiable diseases. As the risks of modifiable diseases remain a major concern in stroke prevention, it is necessary to explore the effect of medication non-adherence on stroke prevention among patients with stroke-related diseases irrespective of the type of medications used. Therefore, this meta-analysis aimed to investigate the impact of medication adherence to major modifiable stroke-related diseases on clinical outcomes among the patients who were never diagnosed with stroke.

## Methods

This meta-analysis was reported according to the Preferred Reporting Items for Systematic Reviews and Meta-Analyses (PRISMA) guideline [23].

### Search strategy

The records were collected from Embase, CINAHL, and PubMed from January 2000 to August 2021. The search keywords are listed as follows: medication adherence, the four major stroke-related diseases (i.e., AF, hyperlipidemia, hypertension, and type 2 diabetes mellitus [T2DM]), medications used to prevent stroke events (e.g., antiplatelets) and to treat the four major diseases (e.g., antidiabetics and antihypertensive agents), stroke-associated outcomes (e.g., stroke occurrence and mortality), relevant clinical terms (e.g., risk factor and prevention) or laboratory testing (e.g., international normalised ratio and haemoglobin A1c [HbA1c]) of stroke, and vascular diseases related to stroke (e.g., cerebrovascular). Those keywords were combined using Boolean operator “AND”, e.g., “medication compliance” AND “antihypertensive” AND “stroke” (Table S1). Some of them used a star (*) to increase the search results. All search restriction strategies used the title and abstract and English as the language. Furthermore, hand searching was performed to look for potential studies from previous related systematic reviews or meta-analyses.

### Eligibility criteria of records

The article records were screened based on the following inclusion criteria: either title or abstract explored the association of medication adherence with stroke-associated outcomes, reported the baseline disease(s) belonging to the aforementioned four major stroke-related diseases, reported risk as hazard ratio (HR) and odds ratio (OR) or frequency of outcomes related to incident stroke (e.g., stroke occurrence and mortality), presented relevant laboratory parameters (e.g., blood glucose), indicated the measurements or tools used to assess medication adherence (e.g., proportion of days covered [PDC] and relevant questionnaires) for outpatients settings, described as observational or experimental study design, and enrolled adults with at least 18 years of age. The articles were excluded if they were either conference abstracts, proceedings, book sections, recommendations of treatments, theses, not written in English, or not available with the information used for quantitative data extraction.

### Study selection

The screening of article records was managed by using EndNote. The potential records were screened out for those irrelevant topics, such as health information, diet, exercise, or qualitative studies, based on the title and abstract. Further checking was conducted based on the retrieved full texts to prepare the final eligible records/studies for further meta-analysis.

### Data extraction

All of the following data related to the research objective in the eligible studies were extracted into the standardised form of a Microsoft Excel file: study characteristics (e.g., first author and publication year), study design (e.g., sample size), clinical information (e.g., baseline of disease and medications), medication adherence/persistence/discontinuation-related information (e.g., definition, threshold, and tool of medication adherence), and study outcomes (e.g., reported risk).

### Definitions of medication adherence and non-adherence

Various measurements/tools or measures were used to categorise the levels of medication adherence to the four major stroke-related diseases and anti-embolism agents, as well as diverse definitions of adherence versus non-adherence (various cut-off points of proportion/scores or discontinuation periods from various diseases and medication types). Thus, we adopted and defined medication adherence more generously, as the literature indicated (Table S2). Levels of medication adherence were defined based on one of these thresholds: (1) the prescription refill adherence (i.e., PDC or medication possession ratio) was ≥ 60%, (2) the total score of the self-reported tool was less than a certain level recommended by the original study (i.e., the total score of the four-item Morisky Medication Adherence Scale < 2), (3) prescription refill records showed patients persistently refilled the same class of medications without an interruption period at least 14 days or longer. In other words, the non-adherent (or non-persistent) to the medications of interest referred the counterpart accordingly, and they were all named as medication non-adherence.

### Quality assessment

Given that all included records obtained from the search findings were either cohort or case–control studies, the Newcastle–Ottawa Scale (NOS) tool was chosen to assess the study quality. The NOS is an eight-item tool used to perform the risk of bias assessment with the subject selection, inter-group comparability, and assurance of either exposure for the included observational studies [24]. Accordingly, two authors worked independently in data extraction and quality assessment, and the third author verified their results. If inconsistencies occurred, further discussions with the third author were carried out until a consensus was reached.

### Outcomes of assessment

The main outcomes were the new occurrence of stroke or related mortality, which were associated with non-adherence to preventive medications, derived from at least two studies [25]. Only those studies with findings exploring the corresponding risks (e.g., HR and OR with 95% confidence interval [CI]), met the defined adherence threshold, and the comparisons between non-adherence versus adherence to the relevant medications of interest were included. Those corresponding risks with adjustment of potential confounding factors were prioritized to be selected in the pooled analysis. For those records with more than one concerned medication that reported their adherence levels, the two-step pooled risk estimates based on the obtained risk estimate and its CI were performed to come up with a “composite pooled risk estimate”. For instance, the pooled estimate of the risk associated with medication non-adherence of dabigatran or rivaroxaban in Borne’s study was used for the next step of the composite pooled risk estimate with the findings obtained from Park and Sohn’s and Jackevicius’s studies (Table S3a). Those steps were similarly used to calculate the further pooled risk estimate of either adherence or non-adherence impact, which were reported from more than two levels of adherence (e.g., low, intermediate, and good adherence). For example, the pooled combination of low and intermediate risk in Kim’s study (2016) was used to calculate the risk of all-cause mortality (Table S3b). Whenever a study reported more than one outcome for an individual subject or adherence measurements at different time points (e.g., at 3, 6, and 12 months), only one outcome for the corresponding adherence measurement, which was related to the most common condition for that specific disease (e.g., ischemic rather than haemorrhagic stroke) and had relatively larger sample size [26], was selected for further analysis.

Given that HR and OR were considered similar to relative risk (RR) for their definitions and interpretations [15], all retrieved risk data of either HR, OR, or RR from individual records were used to perform the pooled risk estimate of the reported outcomes (e.g., stroke occurrence), and were presented as the pooled risk estimate of RR accordingly. The pooled estimates of RRs and their CIs of stroke outcomes which were associated with medication non-adherence were compared in the forest plots and stratified by major disease risk factors or medications of interest.

The pooling analysis was conducted using Cumulative Meta-Analysis (CMA) version 3, a software to perform the meta-analysis [27, 28]. With various measurements or tools to assess the level of medication adherence, study design, disease, and reported medications, we performed a random effect model [25, 29]. The subgroup analyses were performed for various ages, sex, sample sizes, follow-up periods, and study quality to explore the difference in stroke occurrence or mortality risk estimates between non-adherent and adherent patients for the common medications used for stroke prevention. Sensitivity analysis was conducted for each pooled risk estimate as the consequence of medication non-adherence, whenever the leave-one-out approach was performed for those outcomes consisting of more than two records. Only the outcomes with records from at least ten studies were performed for publication bias assessments and subgroup analysis [25]. Subsequently, further sensitivity analysis was performed to assess the consistency of the study findings from the confounding factor effects by removing unadjusted risks from the associated pooled analysis. Further, publication bias assessments were conducted using the funnel plot and Egger’s regression.

## Results

### Study selection

**Fig. 1:**
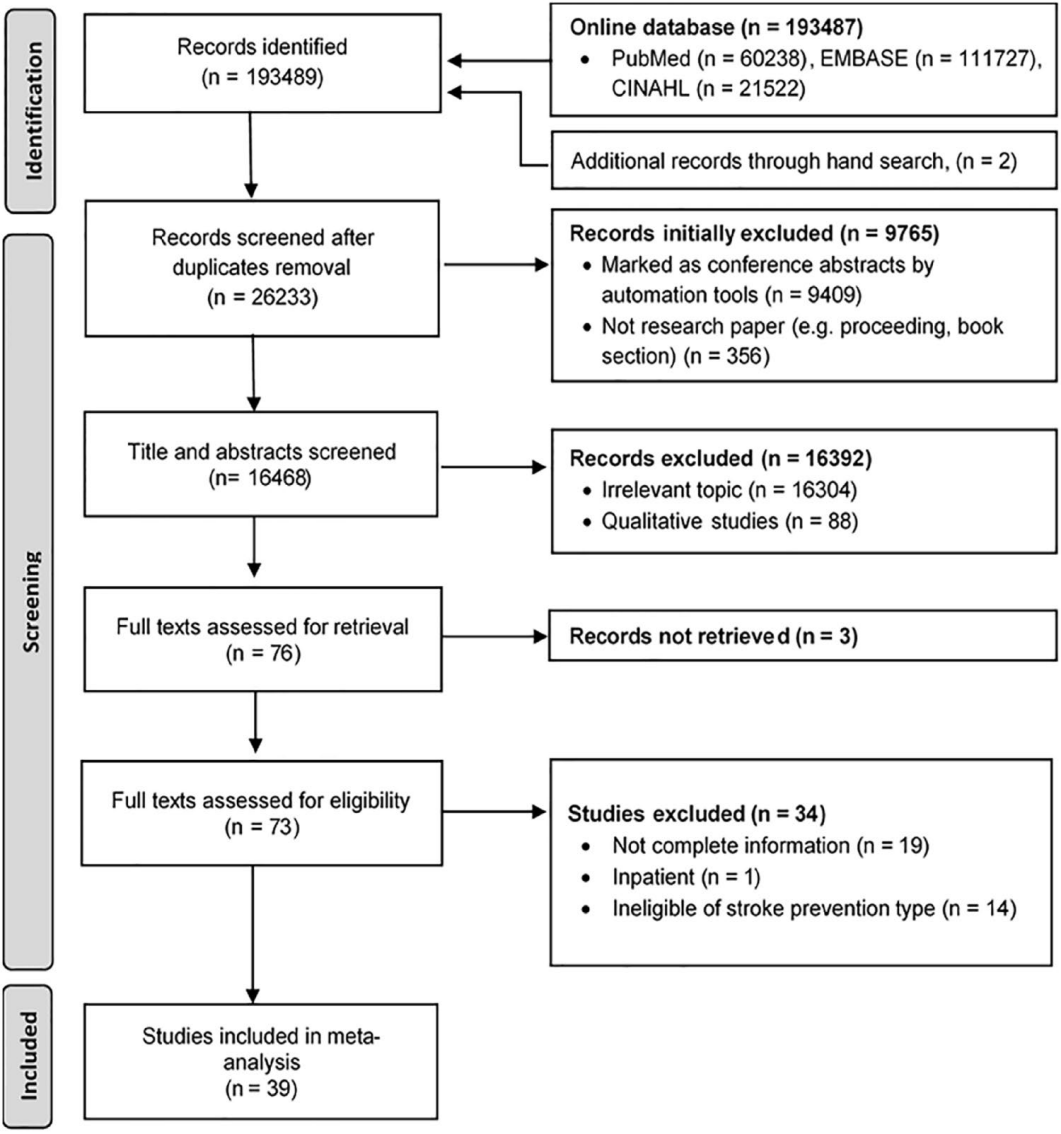
PRISMA flowchart process to select the included studies

Of 73 records with full texts that were assessed for their eligibility, 39 studies were identified for further pooled risk estimates (Fig. 1).

### Quality assessment

There were 7 case–control studies and 32 cohort studies, which were assessed for quality using the NOS. The lowest NOS score was 6, and the highest was 9 in both types of studies. Both results indicated moderate (range of 4–6) and high-quality (range of 7–9) studies (Tables S4a and S4b).

### Characteristic of studies and participants

The characteristics of the 39 identified studies and enrolled participants were summarised in Table 1, and the detailed information was reported in Table S5. The majority of the eligible studies were designed as cohort studies (*n = *32, 82.05%). Over 70% of the studies were designed to assess the prescription refill rate (e.g., PDC) as the measurement of medication adherence or non-adherence. The majority of the studies reported thresholds of medication adherence with either PDC ≥ 80% [30] or > 80% [31], while the others defined non-persistence gap as 14 days or longer, ≥ 45 days, ≥ 60 days, > 3 months, and equal to 100 days or so. Twenty-one studies (53.85%) reported at least a 6-month follow-up period for their participants, starting from the index date of medications or the last date of adherence observation until the relevant outcomes occurred or the end of the follow-up period.

**Table 1:** Characteristics of included studies and participants for the pooled analysis

	*n*	%
Included studies (*n = *39)		
Type of included studies		
Cohort study	32	82.05
Case–control study (nested)	7	17.95
Adherence measurement		
Prescription refill adherence^a^	30	76.92
Persistence or discontinuation^b^	7	17.95
Self-reported	1	2.56
Combination	1	2.56
Follow-up period^c^ (mean/median/per patient)		
≥ 6 months	21	53.85
Partially inform/not reported/only stated as predetermined of follow-up period in the study design	18	46.15
Participants (*n = *2,117,789)		
Female	1,001,276	47.28
Age (mean) ^d^		
< 65 years old	615,916	29.08
≥ 65 years old	754,724	35.64
NA or reported in range across 65 years old	747,149	35.28
Non-adherence (reported from *n = *33 studies)	699,912	39.19^e^
Risk of disease at baseline measurement		
Hypertension	968,089	45.71
Atrial fibrillation	471,482	22.95
Hyperlipidemia	358,263	16.92
Type 2 diabetes mellitus	319,955	15.11
Relevant medication group		
Antihypertensive	968,089	45.71
Antithrombotic	471,482	22.26
Antidiabetic	235,430	11.12
Lipid-lowering agents	383,408	18.10
Combination	58,266	2.75
Not specify the medication name/group	1114	0.05
Measured clinical outcomes (reported from *n = *30 studies)		
Stroke occurrence	71,323	4.80^α^
Either stroke occurrence or death (not specified clearly)	2901	9.68^β^
All-cause mortality	36,151	5.72^¥^
Bleeding	5427	3.95^⁋^

*NA* not available

^a^Medication adherence measured with prescription fill records (i.e., medication possession ratio, proportion of days cover, medication refill adherence, cumulative medication adherence)

^b^Calculating the period of patients adhere to taking medications starts from first use of medication until stops at a certain time

^c^Started from the index date of medications or the last date of adherence observation until the relevant outcome occurred or the end of the follow-up period

^d^Data were collected from studies which reported mean age of their participants

^e^Denominator was the total participants from 33 studies, *n = *1,785,862

^α, β, ¥,^ and ^⁋^: Each denominator of calculation for percentage was retrieved from the total sample size of associated studies, i.e., *α* = 1,486,597 (sample size from relevant articles, *n = *28 studies); *β* = 29,972 (*n = *3 studies); ¥ = 631,569 (*n = *8 studies) and ⁋ = 137,355 (*n = *3 studies)

Of all pooled participants in these 39 studies (*n = *2,117,789), there were relatively fewer female participants (47.08%) than male, and more than one-third (35.64%) reported that they were aged at least 65 years of age. Approximately 40% of the participants (accounted for 33 studies) were reported as non-adherent patients. Of all reported diseases, around 40% of the participants were diagnosed with hypertension, and 20% had AF.

### Association of medication non-adherence with stroke-associated outcomes among the patients who never had a stroke

**Fig. 2:**
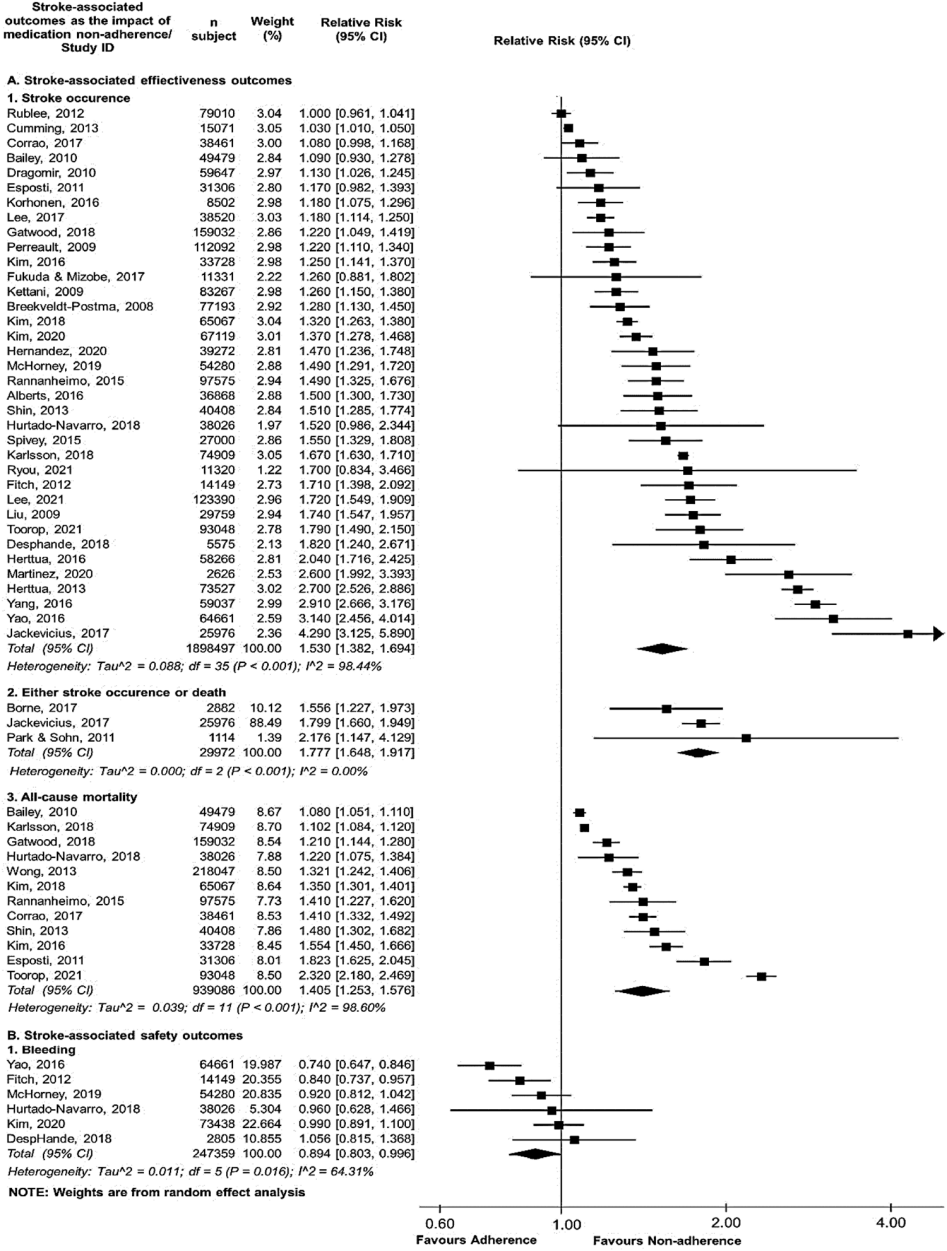
Forest plot of the association between medication non-adherence and effectiveness and safety outcomes upon stroke primary prevention. Aggregate relative risk (RR) was calculated from the data of hazard ratio, odds ratio, and relative risk. *CI* confidence interval

Four pooled risk estimates were reported as consequences of non-adherence to medications of interest. Those consequences covered effectiveness outcomes for stroke occurrence and mortality risks, as well as safety outcomes for bleeding events. Given that stroke occurrence was the most reported outcome, medication non-adherence for primary prevention purposes was significantly associated with the pooled stroke-related negative outcomes but not for the pooled bleeding risk (Fig. 2). Two out of the four pooled relative risks showed considerable heterogeneity (*I*^2^ > 75%), while the other pooled relative risks (i.e., either stroke occurrence or death and bleeding) presented no heterogeneity (*I*^2^ = 0.00%) and substantial heterogeneity (*I*^2^ = 50–90%), respectively.

### Impacts of medication non-adherence on stroke-associated outcomes stratified by disease risks and/or relevant medications of interest

**Fig. 3:**
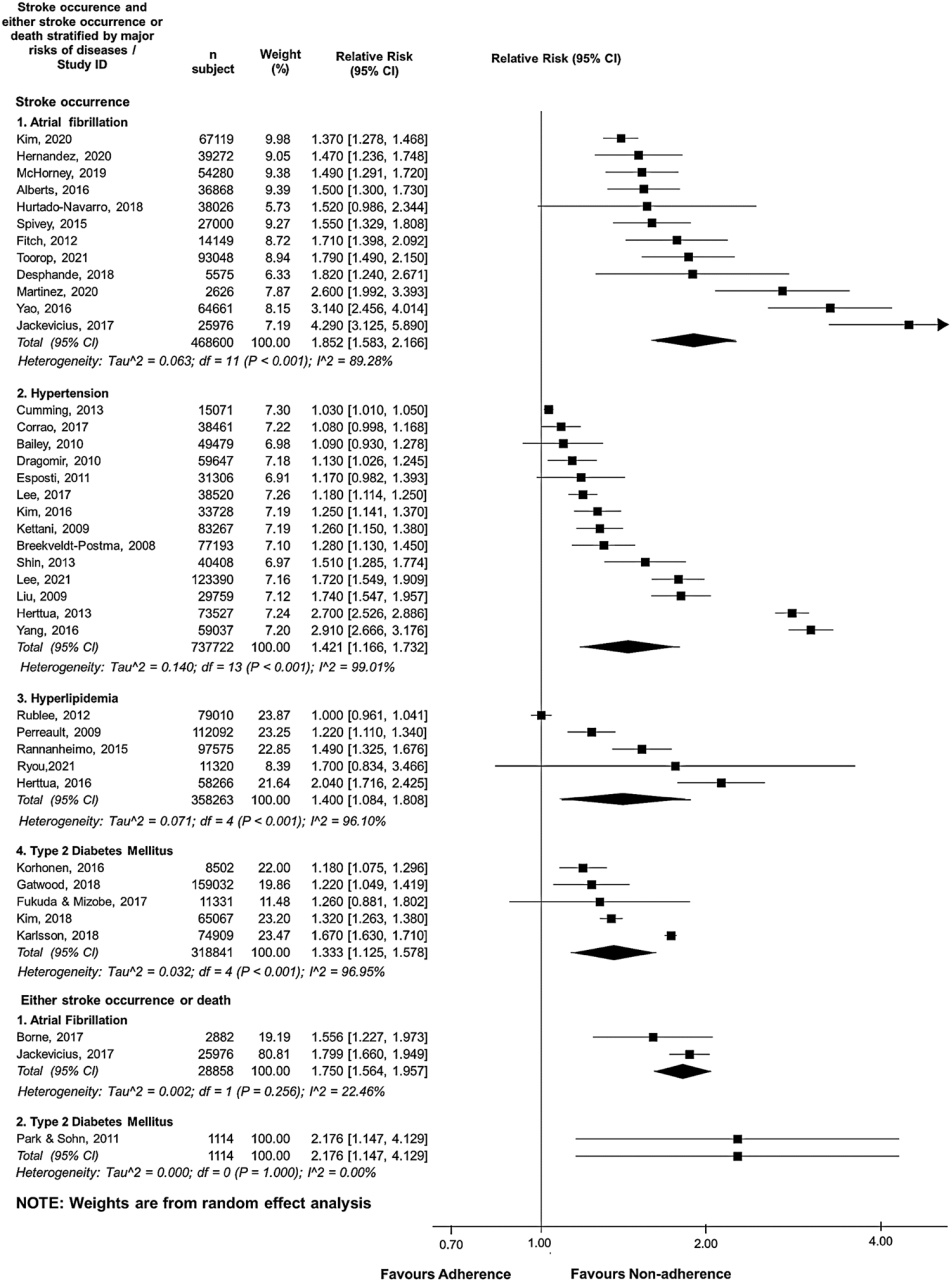
Forest plot of the association between medication non-adherence and effectiveness outcomes associated with stroke occurrence or death, stratified by four major stroke-related diseases in primary stroke prevention. Aggregate relative risk (RR) was calculated from the data of hazard ratio, odds ratio, and relative risk. *CI* confidence interval

**Fig. 4:**
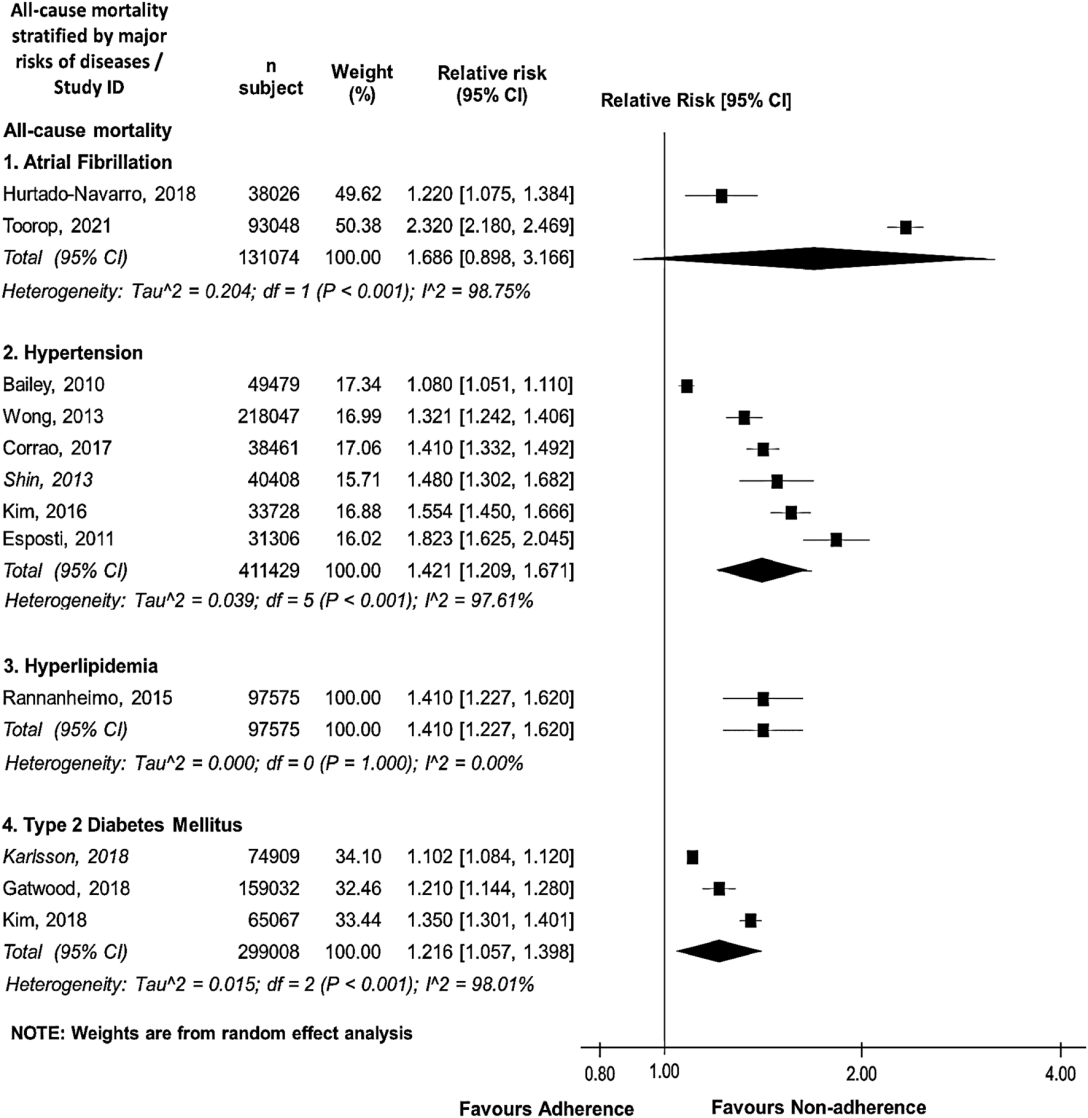
Forest plot of the association between medication non-adherence and effectiveness outcomes associated with all-cause mortality, stratified by four major stroke-related diseases in primary stroke prevention. Aggregate relative risk (RR) was calculated from the data of hazard ratio, odds ratio, and relative risk. *CI* confidence interval

Risks of non-adherence to relevant medication stratified by major modifiable diseases resulted in ten stroke-associated outcomes, comprising four risks related to stroke occurrence (i.e., stroke occurrence in non-adherent patients with AF, hypertensive, hyperlipidemia, and T2DM), two risks of either stroke or death (i.e., either stroke or death in non-adherent patients with AF and T2DM), and four risks of all-cause mortality (i.e., all-cause mortality in non-adherent patients with AF, hypertensive, hyperlipidemia, and T2DM) (Figs. 3 and 4). These outcomes were significantly associated with the increasing pooled stroke-associated negative consequences, except for the pooled RR estimate derived from the patients with AF (Figs. 3 and 4). The non-adherent patients with AF were less likely to encounter bleeding risks in six studies (RR 0.894; 95% CI 0.803–0.996) (Fig. S1).

There were similar patterns of pooled risk estimates, either stratified by relevant medications or risks of diseases, for the stroke-associated outcomes due to medication non-adherence (Tables S6a, S6b, and Fig. S1). In contrast, the pooled risk estimates associated with non-adherence to either antithrombotic or lipid-lowering agents did not significantly increase stroke-associated outcomes [32–35].

### Subgroup analysis, sensitivity analysis, and publication bias

As for the pooled risk of stroke occurrence and all-cause mortality in the subgroup analysis, none of the factors contributed to the change in effect size (Table S7). The results of sensitivity analysis conducted for all potential risks also revealed that all pooled risk estimates were consistent, even if an individual study was removed from analysis at one time (Table S8). Of 39 studies, 33 reported the adjusted risks (e.g., adjusted RR of stroke occurrence, adjusted HR of stroke or death, and adjusted OR of all-cause mortality) (Table S5) after controlling for confounding factors (i.e., age, sex, initial medication, use of cardiovascular co-medications, and disease severity as a baseline data of study [32, 34]). The rest of six studies only reported the number of the first stroke events, number of adherent/non-adherent participants, and sample size, which were used to calculate the associated risks (i.e., OR and RR of stroke occurrence) (Table S5) but could not make further adjustment of the confounding factors. Therefore, a subsequent analysis of the original pooled estimate of 36 studies of stroke occurrence (Fig. 2) was performed by removing the 6 studies with unadjusted risks of stroke occurrence. This analysis resulted in consistent findings (RR 1.558; 95% CI 1.395–1.740) of these 30 studies of stroke occurrence with adjusted risks (Table S8b). As for publication bias, there was an asymmetric funnel plot shape (Figs. S2 and S3); the intercept of risk for stroke occurrence and all-cause mortality upon the Egger’s regressions were 3.700 (*p* = 0.055) and 9.098 (*p* = 0.008), respectively. These indices showed significant publication bias for the outcomes of stroke occurrence and all-cause mortality risks.

## Discussion

This is the first meta-analysis study that examined the association between medication non-adherence to the relevant medications across various modifiable stroke-related diseases and stroke-associated outcomes among patients who were never diagnosed with stroke. Our main findings showed that medication non-adherence among patients with major stroke-related diseases could increase risks of stroke occurrence and all-cause mortality with various significance. Almost all results are consistent when we further stratified analyses by either disease or medications or performed the subgroup and/or sensitivity analyses.

The increasing risks of stroke-related events and all-cause mortality in our study were similar to the previous meta-analyses on assessments of medication adherence to statins [21], antihypertensives [22], and/or cardiovascular medications [15, 16]. Although these studies focused on the risks of stroke-associated outcomes linked to medication adherence, they did not consider the impacts of other disease risk factors. Two studies measured the adherence impact associated with only one baseline disease [18, 19]. In addition, most of the studies above focused only on stroke occurrence or mortality outcomes, not the combination of the outcomes, and were less comprehensive than the current study.

In this study, patients with non-adherence to the relevant medications for primary stroke prevention were all significantly associated with the increased pooled risk estimates of stroke-associated negative outcomes. As demonstrated in a previous meta-analysis study [14], non-adherence could influence medication effects, because patients do not take their medications as prescribed. As a result, the disease progression could not be well controlled, which leads to poor clinical outcomes [36], including all-cause mortality [15].

Further, we demonstrated that non-adherence among patients with AF consistently increased the risk of stroke occurrence and all-cause mortality. Our findings were consistent with the other previous reviews focusing on oral anticoagulants and stroke occurrence [18, 19] and all-cause mortality [18] among patients with AF. In addition, the patients with non-adherence to medications that are used to treat other stroke-related diseases (i.e., hypertension, hyperlipidemia, and T2DM) should also be monitored to avoid the increased risks of stroke-associated negative outcomes. For instance, studies revealed that patients with poor adherence to their medications for AF and hypertension tended to result in incremental risks of stroke events by 3 to 6 times or an average of 3.5 times, compared with patients with high medication adherence [9, 37].

We found that patients with non-adherence to antithrombotic medications (i.e., direct-acting oral anticoagulant [DOAC] and warfarin) were less likely to encounter bleeding risks than those with high medication adherence. This finding meets clinical implications but is inconsistent with the other two previous meta-analyses focusing on DOACs, which indicated that bleeding events were not significantly associated with medication non-adherence [18, 19]. We assumed the discontinuation period may be one of the reasons associated with the reduced bleeding risk [38, 39]. The two corresponding studies addressed that discontinuation of taking DOACs for at least more than 6 months and warfarin for at least more than 12 months would decrease gastrointestinal bleeding risk and major bleeding, respectively. Those patients with fewer risks of stroke (e.g., CHA2DS2-VASc score of 0 or 1), older than 65 years of age, and taking certain medications might be more likely to encounter bleeding events associated with high adherence to antithrombotic agents [38–41].

## Study limitations and strengths

Our meta-analysis has some limitations that should be noted. First, differences in patients’ clinical characteristics (e.g., level of disease severity, healthy lifestyle, stress level, variety of received medication, and prescribed drug regimen) may influence adherence levels and treatment outcomes [14, 42–44]. Second, the main source of study information was administrative data that had restrictions in nature. The data could not be examined precisely regarding actual patient medication adherence, medications taken by patients without prescriptions, and the lack of laboratory testing results (e.g., HbA1c and blood lipid profile) in the majority of databases [45]. Third, the absence of a gold standard of medication measurement [46] and lack of agreement on the adherence threshold [47] may be problematic in evaluating study equality. Fourth, we only identified observational studies, and none of clinical trial studies were eligible for further analysis [43], even if we optimised the search keywords and looked up available databases thoroughly. Fifth, there is a significant problem of publication bias, although we extended searching articles to three databases and included moderate and high-quality studies. We assumed the factors potentially associated with the publication bias in our study include missing unpublished reports and non-English articles [48, 49]. Last but not least, the pooled risk estimates of RR among those non-adherent or adherent patients to the medications of interest were calculated for different types of outcomes and were further stratified upon the stroke-related diseases and types of medications, accordingly. Although the actual differences between HR, OR, or RR from individual records (with various adjusted variables) should not be ignored to calculate the pooled risk estimates, there is no better way to compile the reported risks of HR, OR, or RR, as RR, due to the limited data. In this case, the levels of heterogeneity among the studies for individual outcomes of interest were assessed to avoid overinterpretation. Further subgroup and sensitivity analyses were performed to confirm the robustness of pooled risk estimates for various stroke-associated outcomes.

Even so, there are some strengths in this study. We thoroughly examined the effects of medication non-adherence on all comprehensive stroke-associated outcomes for patients with four major stroke-related diseases rather than ignoring the baselines of major disease risks or focusing specifically on any one stroke-associated outcomes. We also succeeded in including warfarin-related findings in our meta-analysis, given that no other studies have included this point. Lastly, we have a large sample size of studies in some pooled risk analyses, which can increase the power of the findings.

## Conclusion

This meta-analysis demonstrated that non-adherence to medications for four stroke-related diseases and primary stroke prevention would increase stroke occurrence and mortality. These results support the importance of medication adherence to those relevant medications and their impacts on stroke prevention. While some patients find it difficult to take medications as routine to prevent stroke occurrence, more efforts and strategic tactics should be taken to improve patients’ medication adherence from the perspectives of patients, health professionals, and caregivers.

## Supplementary Information

Below is the link to the electronic supplementary material.

Supplementary file1 (DOCX 498 KB)

## Data Availability

The datasets used and/or analysed during the current study are available from the corresponding authors upon reasonable request.

## Abbreviations

AF: Atrial fibrillation
CI: Confidence interval
HR: Hazard ratio
OR: Odds ratio
RR: Relative risk
NOS: Newcastle–Ottawa scale
PDC: Proportion of days covered
T2DM: Type 2 diabetes mellitus
DOAC: Direct-acting oral anticoagulant
DM: Diabetes mellitus
HbA1c: Haemoglobin A1c
PRISMA: Preferred Reporting Items for Systematic Reviews and Meta-Analyses
