# Correction to: Impacts of medication non‑adherence to major modifiable stroke‑related diseases on stroke prevention and mortality: a meta‑analysis

**DOI:** 10.1007/s00415-023-11674-6

**Published:** 2023-03-28

**Authors:** Okti Ratna Mafruhah, Yen-Ming Huang, Hsiang-Wen Lin

**Affiliations:** 1 School of Pharmacy and Graduate Institute, College of Pharmacy, China Medical University, Taichung City, 406040 Taiwan; 2 Department of Pharmacy, Universitas Islam Indonesia, Daerah Istimewa Yogyakarta, 55584 Indonesia; 3 Graduate Institute of Clinical Pharmacy, College of Medicine, National Taiwan University, Taipei City, 100025 Taiwan; 4 School of Pharmacy, College of Medicine, National Taiwan University, Taipei City, 100025 Taiwan; 5 Department of Pharmacy, National Taiwan University Hospital, Taipei City, 100229 Taiwan; 6 Department of Pharmacy, China Medical University Hospital, Taichung City, 404332 Taiwan; 7 Department of Pharmacy System, Outcomes and Policy, College of Pharmacy, University of Illinois at Chicago, Chicago, IL 60612 USA

Correction to: Journal of Neurology 10.1007/s00415-023-11601-9

The original version of this article unfortunately contained a mistake. The corrected details are given below for your reading.

The ORCID (0000-0001-8673-2269) was misplaced to the first author. This number belongs to the second author, Yen-Ming Huang.

There is an omission in the heading of Fig. 3. “orest plot” should be corrected as “Forest plot.”

The original article has been corrected.

