# Supplementary material for: Impacts of medication non-adherence to major modifiable stroke-related diseases on stroke prevention and mortality: a meta-analysis
Source: J Neurol. 2023 Feb 27;270(5):2504–16. doi: 10.1007/s00415-023-11601-9 (PMC9968645; doi:10.1007/s00415-023-11601-9)
Supplement: Supplementary file 1 — Supplementary file1 (DOCX 498 KB) [file 415_2023_11601_MOESM1_ESM.docx]

# **Online supplementary materials**

**Impacts of medication non-adherence to** **major modifiable stroke-related diseases on stroke prevention and mortality: a meta-analysis**

Okti Ratna Mafruhah, Yen-Ming Huang, Hsiang-Wen Lin

Corresponding authors:

Hsiang-Wen Lin: School of Pharmacy and Graduate Institute, College of Pharmacy, China Medical University, Taichung City 406040, Taiwan (e-mail: [hsiangwl@gmail.com](mailto:hsiangwl@gmail.com))

Yen-Ming Huang: Graduate Institute of Clinical Pharmacy, College of Medicine, National Taiwan University, Taipei City 100025, Taiwan ([yenming927@ntu.edu.tw](mailto:yenming927@ntu.edu.tw))

**Table S1.** Keywords used in search records

**Table S2.** Measurements/tools and definitions of medication adherence and persistence

**Table S3a.** Example to come up with the composite pooled risk estimate for the studies with outcomes of either stroke occurrence or death for two medications used for primary stroke prevention

**Table S3b.** Example to come up with the composite pooled risk estimate from more than two levels of medication adherence with outcome of all-cause mortality

**Table S4a.** Critical appraisal with the NOS tool: Case-control study (n = 7)

**Table S4b.** Critical appraisal with the NOS tool: Cohort study (n = 32)

**Table S5.** Characteristics of identified studies used to perform quantitative analysis (n = 39 studies)

**Table S6a.** Relative risk of medication non-adherence impact on stroke-associated outcomes stratified by the group of medications used for major modifiable stroke-related diseases or thrombosis prevention

**Table S6b.** Relative risk of medication non-adherence impact on stroke-associated outcomes without stratification of medication used for major modifiable diseases or thrombosis prevention

**Table S7**. Subgroup analysis of medication non-adherence impact associated with major modifiable diseases on stroke events and all-cause mortality risks

**Table S8a.** Sensitivity analysis of stroke associated-outcomes as a consequence of medication non-adherence when one study was removed at one time

**Table S8b.** Sensitivity analysis of stroke occurrence as a consequence of medication non-adherence after removing 6 unadjusted risks from the pooled analysis

**Fig. S1** Pooled risk estimates of the safety outcome associated with non-adherence versus adherence to medications among patients taking medications for primary stroke prevention without disease or medication stratification

**Fig. S2** Funnel plot of the relative risk of the impact of medication non-adherence associated major modifiable stroke-related diseases on stroke occurrence

**Fig. S3** Funnel plot of the relative risk of the impact of medication non-adherence associated with major modifiable stroke-related diseases on all-cause mortality

**Table S1.** Keywords used in search records

| **Online database** | **Group keywords** | **Keywords** |
| --- | --- | --- |
| **Pubmed** | **Keywords group A** (cover medication adherence terms) | “Medication adherence” (tiab), “Medication compliance”(tiab), “Medication persistence”(tiab), Adherence(tiab), Persistence(tiab), Compliance(tiab), Concordance(tiab) |
|  | **Keywords group B** (cover four disease of stroke risk factors, associated medication, relevant clinical test terms, and stroke outcomes) | antihypertensive*(tiab), angiotensin-converting enzyme inhibitor*(tiab), adrenergic beta-antagonists(tiab), angiotensin receptor blocker*(tiab), beta blocker*(tiab), calcium channel blocker*(tiab), thiazide*(tiab), hypoglycemic*(tiab), antidiabetic*(tiab), insulin(tiab), metformin(tiab), pioglitazone(tiab), antiplatelet*(tiab), aspirin(tiab), clopidogrel(tiab), warfarin(tiab), anticoagulant*(tiab), NOAC*(tiab), DOAC*(tiab), dabigatran(tiab), rivaroxaban(tiab), Apixaban(tiab), edoxaban(tiab), cholesterol-lowering*(tiab), antihyperlipidem*(tiab), hydroxymethylglutaryl-coa reductase inhibitor*(tiab), statin*(tiab), "risk factor*"(tiab), prevention(tiab), control(tiab), blood pressure(tiab), hypertension(tiab), atrial fibrillation(tiab), INR(tiab), hypercholesterolem*(tiab), hyperlipidem*(tiab), lipoprotein*(tiab), diabetes*(tiab), HbA1c(tiab), glucose*(tiab), adverse*(tiab), bleeding*(tiab), hypotension(tiab), hypoglycem*(tiab), thrombocytopen*(tiab), liver function tests(tiab), LFTs(tiab), myopathy(tiab), rhabdomyolysis(tiab), thromboemboli*(tiab), Occurrence(tiab), recurrence(tiab), hospitalization*(tiab), admission*(tiab), mortality(tiab), death*(tiab), emergency*(tiab), visit*(tiab) |
|  | **Keywords group C** (cover stroke related terms) | Stroke*(tiab), Cerebrovascular(tiab), Cardiovascular(tiab) |
| **Embase** | **Keywords group A** (cover medication adherence terms) | ‘Medication adherence’:ab,ti; ‘Medication compliance’:ab,ti; ‘Medication persistence’:ab,ti; Adherence:ab,ti; Persistence:ab,ti; Compliance:ab,ti; Concordance:ab,ti |
|  | **Keywords group B** (cover four disease of stroke risk factors, associated medication, relevant clinical test terms, and stroke outcomes) | antihypertensive*:ab,ti; angiotensin-converting enzyme inhibitor*:ab,ti; adrenergic beta-antagonists:ab,ti; angiotensin receptor blocker*:ab,ti; beta blocker*:ab,ti; calcium channel blocker*:ab,ti; thiazide*:ab,ti; hypoglycemic*:ab,ti; antidiabetic*:ab,ti; insulin:ab,ti; metformin:ab,ti; pioglitazone:ab,ti; antiplatelet*:ab,ti; aspirin:ab,ti; clopidogrel:ab,ti; warfarin:ab,ti; anticoagulant*:ab,ti; NOAC*:ab,ti; DOAC*:ab,ti; dabigatran:ab,ti; rivaroxaban:ab,ti; Apixaban:ab,ti; edoxaban:ab,ti; cholesterol-lowering*:ab,ti; antihyperlipidem*:ab,ti; hydroxymethylglutaryl-coa reductase inhibitor*:ab,ti; statin*:ab,ti; "risk factor*":ab,ti; prevention:ab,ti; control:ab,ti; blood pressure:ab,ti; hypertension:ab,ti; atrial fibrillation:ab,ti; INR:ab,ti; hypercholesterolem*:ab,ti; hyperlipidem*:ab,ti; lipoprotein*:ab,ti; diabetes:ab,ti; HbA1c:ab,ti; glucose*:ab,ti; adverse*:ab,ti; bleeding*:ab,ti; hypotension:ab,ti; hypoglycem*:ab,ti; thrombocytopen*:ab,ti; liver function tests:ab,ti ; LFTs:ab,ti; myopathy:ab,ti; rhabdomyolysis:ab,ti; thromboemboli*:ab,ti; Occurrence:ab,ti; recurrence:ab,ti; hospitalization*:ab,ti; admission*:ab,ti; mortality:ab,ti; death*:ab,ti; emergency*:ab,ti; visit*:ab,ti |
|  | **Keywords group C** (cover stroke related terms) | Stroke*:ab,ti; Cerebrovascular:ab,ti; cardiovascular:ab,ti |
| **CINAHL** | **Keywords group A** (cover medication adherence terms) | TI “Medication adherence” OR AB “Medication adherence”, TI “Medication compliance” OR AB “Medication compliance”, TI “Medication persistence” OR AB “Medication persistence”, TI Adherence OR AB Adherence, TI Persistence OR AB Persistence, TI Compliance OR AB Compliance, TI Concordance OR AB Concordance |
|  | **Keywords group B** (cover four disease of stroke risk factors, associated medication, relevant clinical test terms, and stroke outcomes) | TI antihypertensive* OR AB antihypertensive*, TI angiotensin-converting enzyme inhibitor* OR AB angiotensin-converting enzyme inhibitor*, TI adrenergic beta-antagonists OR AB adrenergic beta-antagonists, TI angiotensin receptor blocker* OR AB angiotensin receptor blocker*,  TI beta blocker* OR AB beta blocker*, TI calcium channel blocker* OR AB calcium channel blocker*, TI thiazide* OR AB thiazide*, TI hypoglycemic* OR AB hypoglycemic*, TI antidiabetic* OR AB antidiabetic*, TI insulin OR AB insulin, TI metformin OR AB metformin,  TI pioglitazone OR AB pioglitazone, TI antiplatelet* OR AB antiplatelet*, TI aspirin OR AB aspirin, TI clopidogrel OR AB clopidogrel, TI warfarin OR AB warfarin, TI anticoagulant* OR AB anticoagulant*, TI NOAC* OR AB NOAC*, TI DOAC* OR AB DOAC*, TI dabigatran OR AB dabigatran, TI rivaroxaban OR AB rivaroxaban, TI Apixaban OR AB Apixaban, TI edoxaban OR AB edoxaban, TI cholesterol-lowering* OR AB cholesterol-lowering*, TI antihyperlipidem* OR AB antihyperlipidem*, TI hydroxymethylglutaryl-coa reductase inhibitor* OR AB hydroxymethylglutaryl-coa reductase inhibitor*, TI statin* OR AB statin*, TI "risk factor*" OR AB "risk factor*", TI prevention OR AB prevention, TI control OR AB control, TI blood pressure OR AB blood pressure, TI hypertension OR AB hypertension, TI atrial fibrillation OR AB atrial fibrillation, TI INR OR AB INR, TI hypercholesterolem* OR AB hypercholesterolem*, TI hyperlipidem* OR AB hyperlipidem*, TI lipoprotein* OR AB lipoprotein*, TI diabetes OR AB diabetes, TI HbA1c OR AB HbA1c, TI glucose* OR AB glucose*, TI adverse* OR AB adverse*, TI bleeding* OR AB bleeding*, TI hypotension OR AB hypotension, TI hypoglycem* OR AB hypoglycem*, TI thrombocytopen* OR AB thrombocytopen*, TI liver function tests OR AB liver function tests, TI LFTs OR AB LFTs, TI myopathy OR AB myopathy, TI rhabdomyolysis OR AB rhabdomyolysis, TI thromboemboli* OR AB thromboemboli*, TI Occurrence OR AB Occurrence,  TI recurrence OR AB recurrence, TI hospitalization* OR AB hospitalization*, TI admission* OR AB admission*, TI mortality OR AB mortality, TI death* OR AB death*, TI emergency* OR AB emergency*, TI visit* OR AB visit* |
|  | **Keywords group C** (cover stroke related terms) | TI Stroke* OR AB Stroke*,  TI Cerebrovascular OR AB Cerebrovascular,  TI Cardiovascular OR AB Cardiovascular |

Note. (1) the star (*) was determined after optimization of keywords before use. (2) (tiab) or ab,ti or TI AB were refer to Title/Abstract. (3) Keywords for searching are composed from group A + B + C, e.g., Pubmed: “Medication adherence”(ti/ab) AND antihypertensive*(ti/ab) AND stroke*(ti/ab); Embase: ‘medication adherence’:ab,ti AND antihypertensive*:ab;ti AND stroke*:ab,ti; CINAHL: ( TI “Medication adherence” OR AB “Medication adherence” ) AND ( TI antihypertensive* OR AB antihypertensive* ) AND ( TI Stroke* OR AB Stroke* )

**Table S2. Measurements/tools and definitions of medication adherence and persistence**

| **Adherence measurements/tool(s)** | **Definition and cut-off points of medication adherence** |
| --- | --- |
| **Measurement used to assess prescription refill based on prescription databases** | |
| Proportion of Days Covered (PDC) | PDC: the total number of days a patient had a medication available divided by the total number of days of study participation [1].  High adherence or adherent was defined if PDC ≥ 60% [2-4]. |
| Medication Possession Ratio (MPR) | MPR: the ratio of total number of days’ supply of medication dispensed to the number of days of study participation per patient [5].  MPR values of >80% or ≥80% were defined as high adherence or adherent [5]. |
| Medication Refill Adherence (MRA) | MRA: the total of days’ supply for all qualifying drug classes divided by the total number of days from the first to the last refill in the study interval [6].  MRA can be used to calculate medication combinations. MRA produces the identical result of adherence as it similarly calculates MPR [6].  MRA values reached > 80% or ≥ 80%, defined as high adherence or adherent [6]. |
| Cumulative Medication Adherence (CMA) | CMA: the sum of the days of medication supplied (obtained over a series of intervals) divided by the total treatment duration (days) from the beginning to the end of the time prescription period dispensed [7].  CMA in Kim et al. (2016) [7] study refers to CMA in Steiner et al. (1997) [8] as Continuous Measure of Medication Acquisition.  CMA values of > 80% or ≥ 80% defined as high adherence or adherent. |
| Compliance rate | Compliance rate: the cumulative number of months with prescribed medication up to time t divided by the total number of months since the initial prescription up to time t [9] Compliance rate values of > 70% was defined as high adherence or adherent. |
| **Measure used to assess persistence or discontinuation based on prescription databases** | |
| Persistence or discontinuation of therapy | Persistence: the duration of time between initiation (start to take medication) and discontinuation of treatment. [10]  Patient is defined as persistent whenever they continuously taking medication in same class/group of medication start from the index date until the end of adherence/persistence observation period or end of study or death (which comes first) without a gap (discontinue/stop taking medication). Non-persistence gap of therapy or discontinuation period was defined as the interruption period between two dispensing, for at least equal to 14 days [11], which was the shortest gap period of the included studies. |
| **Self-reported measure** |  |
| The 4-item Morisky Medication Adherence Scale (MMAS-4) | MMAS-4 = a validated tool to measure medication adherence based on self-reported of patients, with response of each item as “yes” or “no”.  Total score of MMAS-4 which categorized as with adherence was less than 2 [12]. |

**Table S3a.** Example to come up with the composite pooled risk estimate for the studies with outcomes of either stroke occurrence or death for two medications used for primary stroke prevention

| **Step 1:** to perform the pooled estimate based on the original data obtained from the risk of non-adherence for different medication in one study | | | |
| --- | --- | --- | --- |
| **Output of risk estimate in step 1 in CMA analysis** | | | |
| **Study** | **Original data** | **HR (95% CI)** | **Relative Weight*** |
| Borne at al., 2017 | Dabigatran | 1.540 (1.200, 1.970) | 91.56 |
|  | Rivaroxaban | 1.740 (0.770, 3.940) | 8.44 |
| **Pooled risk estimate (fixed effect model)** | | **1.556 (1.227, 1.972)** |  |

| **Step 2:** to perform the pooled risk estimate of medication non-adherence based on either the original data or the prior pooled estimate for different studies or records | | | |
| --- | --- | --- | --- |
| **Output of risk estimate in step 2 in CMA analysis** | | | |
| **Study** | **HR (95% CI)** | **P value** | **Relative weight*** |
| **Borne et al., 2017** | **1.556 (1.227, 1.972)** | **< 0.001** | **88.49** |
| Jackevicius et al, 2017 | 1.799 (1.660, 1.949) | < 0.001 | 1.39 |
| Park & Sohn, 2011 | 2.176 (1.147, 4.130) | 0.017 | 10.12 |
| **Composite pooled risk estimate**  **(random effect model)** | 1.777 (1.648, 1.917) | < 0.001 |  |

^*^ The corresponding relative weight in each row was derived from the variance of the corresponding finding in that study.

# **Table S3b.** Example to come up with the composite pooled risk estimate from more than two levels of medication adherence with outcome of all-cause mortality

| **Step 1:** to perform the pooled estimate based on the original data obtained from the risk of non-adherence for different medication in one study | | | |
| --- | --- | --- | --- |
| **Output of risk estimate in step 1 in CMA analysis** | | | |
| **Study** | **Original data** | **HR (95% CI)** | **Relative Weight*** |
| Kim et al., 2016^#^ | Intermittent | 1.390 (1.260, 1.530) | 51.51 |
|  | Poor | 1.750 (1.580, 1.930) | 48.49 |
| **Pooled risk estimate (fixed effect model)** | | **1.554 (1.450, 1.666)** |  |

^#^ Kim et al. (2016) has three levels of medication adherence, i.e., good, intermittent, and poor adherence. Good adherence was the reference to calculate the risk of intermittent and poor adherence in the original data.

| **Step 2**: to perform the pooled risk estimate of medication non-adherence based on either the original data or the prior pooled estimate for different studies or records | | | |
| --- | --- | --- | --- |
| **Output of risk estimate in step 2 in CMA analysis** | | | |
| **Study** | **HR (95% CI)** | **P value** | **Relative weight*** |
| Bailey et al., 2010 | 1.080 (1.051, 1.110) | < 0.001 | 8.67 |
| Esposti et al., 2011 | 1.823 (1.625, 2.045) | < 0.001 | 8.01 |
| Wong et al., 2013 | 1.321 (1.242, 1.406) | < 0.001 | 8.50 |
| **Kim et al., 2016** | **1.554 (1.450, 1.666)** | **< 0.001** | **8.45** |
| Karlsson et al., 2018 | 1.102 (1.084, 1.120) | < 0.001 | 8.70 |
| Toorop et al., 2021 | 2.320 (2.180, 2.469) | < 0.001 | 8.50 |
| Kim et al., 2018 (a. Primary) | 1.350 (1.301, 1.401) | < 0.001 | 8.64 |
| Rannanheimo et al., 2015 | 1.410 (1.227, 1.620) | < 0.001 | 7.73 |
| Shin et al., 2013 | 1.480 (1.302, 1.682) | < 0.001 | 7.86 |
| Hurtado-Navarro et al., 2018 | 1.220 (1.075, 1.384) | 0.002 | 7.88 |
| Corrao et al., 2017 | 1.410 (1.332, 1.492) | < 0.001 | 8.53 |
| Gatwood et al., 2018 | 1.210 (1.144, 1.280) | < 0.001 | 8.54 |
| **Composite pooled risk estimate**  **(random effect model)** | 1.405 (1.253, 1.576) | < 0.001 |  |

^*^ The corresponding relative weight in each row was derived from the variance of corresponding finding in that study.

# **Table S4a.** Critical appraisal with the NOS tool: Case-control study (n = 7)

| **No** | **Study ID** | **Selection** | | | | **Comparability of cases and controls on the basis of the design or analysis** | **Exposure** | | | **Total score** |
| --- | --- | --- | --- | --- | --- | --- | --- | --- | --- | --- |
|  |  | **Is the case definition adequate?** | **Representativeness of the cases** | **Selection of controls** | **Definition of controls** |  | **Ascertainment of exposure** | **Same method of ascertainment for cases and controls** | **Non-response rate** |  |
| 1 | Kettani et al., 2009 | 0 | 1 | 1 | 1 | 2 | 1 | 1 | 0 | 7 |
| 2 | Perreault et al., 2009 | 0 | 1 | 1 | 1 | 2 | 1 | 0 | 1 | 7 |
| 3 | Herttua et al., 2013 | 0 | 1 | 1 | 1 | 2 | 1 | 0 | 1 | 7 |
| 4 | Herttua et al., 2016 | 0 | 1 | 1 | 1 | 1 | 1 | 0 | 1 | 6 |
| 5 | Korhonen et al., 2016 | 1 | 1 | 1 | 1 | 2 | 1 | 1 | 1 | 9 |
| 6 | Corrao et al., 2017 | 0 | 1 | 1 | 1 | 1 | 1 | 0 | 1 | 6 |
| 7 | Martinez et al., 2020 | 0 | 1 | 1 | 1 | 1 | 1 | 1 | 1 | 7 |

# **Table S4b.** Critical appraisal with the NOS tool: cohort study (n = 32)

| **No** | **Study ID** | **Selection** | | | | **Comparability of cohorts on the basis of the design or analysis** | **Outcome** | | | **Total score** |
| --- | --- | --- | --- | --- | --- | --- | --- | --- | --- | --- |
|  |  | **Representativeness of the sample** | **Selection of the nonintervention cohort** | **Ascertainment of intervention** | **Demonstration that outcome of interest was not represent at start of study** |  | **Assessment of outcome** | **Was follow up long enough for outcomes to occur** | **Adequacy of follow up of cohorts** |  |
| 1 | Breekveldt-Postma et al., 2008 | 1 | 1 | 1 | 1 | 0 | 1 | 1 | 1 | 7 |
| 2 | Liu et al., 2009 | 1 | 1 | 1 | 1 | 1 | 1 | 1 | 1 | 8 |
| 3 | Bailey et al., 2010 | 1 | 1 | 1 | 1 | 2 | 1 | 1 | 1 | 9 |
| 4 | Dragomir et al., 2010 | 1 | 1 | 1 | 1 | 2 | 1 | 1 | 1 | 9 |
| 5 | Esposti et al., 2011 | 1 | 1 | 1 | 1 | 1 | 1 | 1 | 1 | 8 |
| 6 | Park & Sohn, 2011 | 1 | 1 | 1 | 1 | 1 | 1 | 1 | 0 | 7 |
| 7 | Fitch et al., 2012 | 1 | 1 | 1 | 0 | 1 | 1 | 1 | 1 | 7 |
| 8 | Rublee et al., 2012 | 1 | 1 | 1 | 1 | 2 | 1 | 1 | 1 | 9 |
| 9 | Cummings et al., 2013 | 1 | 1 | 1 | 0 | 1 | 1 | 1 | 0 | 6 |
| 10 | Wong et al., 2013 | 1 | 1 | 1 | 1 | 1 | 1 | 1 | 1 | 8 |
| 11 | Shin et al., 2013 | 1 | 1 | 1 | 1 | 2 | 1 | 1 | 1 | 9 |
| 12 | Spivey et al., 2015 | 1 | 1 | 1 | 1 | 2 | 1 | 1 | 1 | 9 |
| 13 | Rannanheimo et al., 2015 | 1 | 1 | 1 | 1 | 2 | 1 | 1 | 1 | 9 |
| 14 | Kim et al., 2016 | 1 | 1 | 1 | 1 | 2 | 1 | 1 | 1 | 9 |
| 15 | Yao et al., 2016 | 1 | 1 | 1 | 1 | 2 | 1 | 1 | 1 | 9 |
| 16 | Alberts et al., 2016 | 1 | 1 | 1 | 1 | 2 | 1 | 1 | 0 | 8 |
| 17 | Yang et al., 2016 | 1 | 1 | 1 | 1 | 2 | 1 | 1 | 1 | 9 |
| 18 | Borne et al., 2017 | 1 | 1 | 1 | 0 | 0 | 1 | 1 | 1 | 6 |
| 19 | Lee et al., 2017 | 1 | 1 | 1 | 1 | 1 | 1 | 1 | 1 | 8 |
| 20 | Jackevicius et al., 2017 | 1 | 1 | 1 | 0 | 2 | 1 | 1 | 1 | 8 |
| 21 | Fukuda & Mizobe, 2017 | 1 | 1 | 1 | 1 | 2 | 1 | 1 | 1 | 9 |
| 22 | Desphande et al., 2018 | 1 | 1 | 1 | 1 | 2 | 1 | 1 | 1 | 9 |
| 23 | Karlsson et al., 2018 | 1 | 1 | 1 | 0 | 2 | 1 | 1 | 1 | 8 |
| 24 | Gatwood et al., 2018 | 1 | 1 | 1 | 1 | 2 | 1 | 1 | 1 | 9 |
| 25 | Kim et al., 2018 | 1 | 1 | 1 | 1 | 2 | 1 | 1 | 0 | 8 |
| 26 | Hurtado-Navarro et al., 2018 | 1 | 1 | 1 | 1 | 2 | 1 | 1 | 1 | 9 |
| 27 | McHorney et al., 2019 | 1 | 1 | 1 | 1 | 2 | 1 | 1 | 1 | 9 |
| 28 | Kim et al., 2020 | 1 | 1 | 1 | 0 | 2 | 1 | 1 | 1 | 8 |
| 29 | Hernandez et al., 2020 | 1 | 1 | 1 | 1 | 2 | 1 | 1 | 1 | 9 |
| 30 | Toorop et al., 2021 | 1 | 1 | 1 | 1 | 1 | 1 | 1 | 1 | 8 |
| 31 | Ryou et al.,2021 | 1 | 1 | 1 | 1 | 2 | 1 | 1 | 1 | 9 |
| 32 | Lee et al., 2021 | 1 | 1 | 1 | 1 | 2 | 1 | 1 | 0 | 8 |

# **Table S5.** Characteristics of identified studies used to perform quantitative analysis (n = 39 studies)

| **Study name, year** | **Country; Study design; sample size (% female)** | **Follow-up period definition and length of follow-up (years)** | **Age (years old)** | **Major modifi-able disease investi-gated** | **Group of medica-tion investi-gated** | **Defined medication adherence or persistence which was related to the outcome of interest** | **Outcome reported** |
| --- | --- | --- | --- | --- | --- | --- | --- |
| Breekveldt-Postma et al., 2008 [13] | Netherlands; Cohort;  77,193 (59.90) | Refers to outcome follow-up, i.e., 20–25% of sample = 3–5, about 20–25% = 5-10, and others = NA | Mean = 55.2 ± 15.3 | HT | AHT | The number of days of continuous use of medication from the index date, uninterrupted durations between two dispensing < 60 days. | RR of stroke |
| Kettani et al., 2009 [5] | Canada; Nested case-control;  83,267 (62.70) | Start = index date,  End = relevant event or end of follow-up;  Mean = 3.17 ± 1.68 | Mean = 65 | HT | AHT | MPR ≥ 80% | OR^*^ of stroke |
| Liu et al., 2009 [9] | Taiwan; Cohort;  29,759 (48.20) | Start = index date,  End = relevant event or end of follow-up;  Mean = 3.23 ± 1.75 | Mean = 55.3 ± 12.5 | HT (newly diagnosed, uncomplicated) | AHT | Compliance rate ≥ 70% | HR of stroke |
| Perreault et al., 2009 [14] | Canada; Nested case-control;  112,092 (59.00) | Start = index date,  End = relevant event or end of follow-up;  Mean = 2.95 | Mean, all = NA; low adherence = 62.5 ± 10; high adherence = 63.5 ± 9 | HL | LLA | MPR ≥ 80% | OR^*^ of stroke |
| Bailey et al., 2010 [6] | United States; Cohort;  49,479 (67.71) | Start = index date,  End = relevant event or end of follow-up;  Mean = 2.70 | Mean = 48.5 | HT | AHT | MRA ≥ 80% | HR of stroke, HR of ACM |
| Dragomir et al., 2010 [15] | Canada; Cohort;  56,896 (64.36) | Start = index date,  End = relevant event or end of 3-year predetermined follow-up in the study design;  Each of patient = 3 | Mean, all = NA; non-adherent= 64.7 ± 10; adherent= 65.2 ± 9 | HT | AHT | MPR ≥ 80% | OR of stroke |
| Esposti et al., 2011 [1] | Italy;  Cohort;  31,306 (62.00) | Start = index date,  End = relevant event or end of follow-up;  Median = 1.9, maximum = 3.5 | Mean = 60.2 ± 14.5 | HT (newly treated) | AHT | PDC > 80% | HR of stroke, HR of ACM |
| Park & Sohn, 2011 [16] | South Korea; Cohort;  1,114 (33.39) | Started from index date, no further information of end point of follow-up. The study mentioned the stroke event of cerebrovascular was 3.82 | Mean = NA | T2DM (newly treated) | NA | CMA ≥ 80% | HR of stroke or death |
| Fitch et al., 2012 [17] | United States; Cohort;  14,149 (58.70) | Start = index date,  End = relevant event or end of 1-year predetermined follow-up in the study design;  Follow-up of each patient = 1 | Mean = 79.3 ± 8.3 | NVAF | AT | PDC ≥ 80% | OR of stroke, OR of bleeding |
| Cummings et al., 2013 [12] | United States; Cohort;  15,071 (56.07) | Start = index date,  End = relevant event or end of follow-up;  Mean = 4.9 (SD NA) | Mean ≥ 65 | HT | AHT | MMAS-4 items,  total score < 2 | HR of stroke |
| Wong et al., 2013 [18] | Hongkong; Cohort;  218,047 (54.90) | Start = index date,  End = relevant event or end of 5-year predetermined follow-up in the study design;  Follow-up of each patient/mean/median reported = NA | Mean = NA | HT | AHT | PDC ≥ 80% | HR of ACM |
| Herttua et al., 2013 [19] | Finlandia; Nested case-control;  73,527 (57.00) | Start = index date,  End = relevant event or end of follow-up;  Mean, all = NA, mean of cases = 7.0 ± 3.8; control=1.7 ± 1.8 | Mean, all = NA; cases = 73.0 ± 10.7; controls = 65.1 ± 12.1 | HT | AHT | PDC > 80% | OR of stroke |
| Shin et al., 2013 [20] | Korea;  Cohort;  40,408 (49.70) | Start = index date,  End = relevant event or end of 5-year predetermined follow-up in the study design;  Follow-up of each patient/mean/median reported = NA | Mean = 51 ± 13 | HT | AHT | MPR ≥ 80% | HR of stroke, HR of ACM |
| Spivey et al., 2015 [21] | United States; Cohort;  27,000 (41.60) | Start = index date,  End = relevant event or end of 1-year predetermined follow-up in the study design;  Follow-up of each patient/mean/median reported = NA | Mean = 71.87 ± 11.09 | NVAF | AT | Medication therapy without a gap ≥ 45 days between the end date of the former prescription and the start date of the current prescription; non-persistence gap (discontinuation period) of therapy ≥ 45 days | HR of stroke |
| Rannanheimo et al., 2015 [22] | Finlandia; Cohort;  97,575 (56.00) | Start = index date,  End = relevant event or end of 3-year predetermined follow-up in the study design;  Follow-up of each patient/mean/median reported = NA | Mean = 59.2 ± 7.8 | HL | LLA | PDC ≥ 80% | HR of stroke, HR of ACM |
| Kim et al., 2016 [7] | South Korea; Cohort;  33,728 (53.40) | Start = index date,  End = relevant event or end of follow-up;  Mean = 5, extended ≤ 7 | Mean = NA | HT (newly treated) | AHT | CMA ≥ 80% | HR of stroke, HR of ACM |
| Korhonen et al., 2016 [23] | Finlandia; Nested case-control;  8,502 (37.10) | Start = index date,  End = relevant event or end of follow-up;  Mean = 3.9 | Mean, all = NA; case = 64.20; control = 64.10 | T2DM | LLA | PDC ≥ 80% | OR^*^ of stroke |
| Yao et al., 2016 [24] | United States; Cohort;  64,661 (43.80) | Start = index date,  End = relevant event or end of follow-up;  Median (IQR) = 1.1 (0.5–2.0) | Median (IQR) = 73 (64-80) | AF | AT | PDC ≥ 80% (to calculate n of non-adherence participants); continuously taking medication and discontinuation period was ≥ 6 months (to calculate risk reported) | HR of stroke, HR of bleeding |
| Herttua et al., 2016 [25] | Finlandia; Nested case-control;  58,266 (53.81) | Start = index date,  End = relevant event or end of follow-up;  Mean, all = NA; non-cases = 5.5 ± 3.4; fatal = 4.7 ± 3.0 | Mean, all = NA; non-cases: 64.3 ± 11.2; fatal: 72.8 ± 9.7 | HL | LLA and/or AHT | PDC ≥ 80% | OR of stroke |
| Alberts et al., 2016 [26] | United States; Cohort;  36,868 (44.95) | Start = index date,  End = relevant event or end of 1-year predetermined follow-up in the study design;  Follow-up of each patient/mean/median reported = NA | Mean = NA | AF | AT | PDC ≥ 80% | HR of stroke |
| Yang et al., 2016 [27] | United States; Cohort;  59,037 (67.30) | Start = index date,  End = relevant event or end of 3-year predetermined follow-up in the study design;  Follow-up of each patient/mean/median reported = NA | Mean = 50.3 | HT | AHT | MPR ≥ 80% | RR^*^ of stroke |
| Borne et al., 2017 [28] | United States; Cohort;  2,882 (3.10) | Start = index date,  End = relevant event or end of follow-up;  Mean = 1.83 ± 1.18 and  median (IQR) = 1.59 (1.06 - 2.56) | Mean = 67.4 ± 9.5 | AF | AT | PDC ≥ 80% | HR of stroke or death |
| Lee et al., 2017 [29] | South Korea; Cohort;  38,520 (45,80) | Start = index date,  End = relevant event or end of 5-year predetermined follow-up in the study design;  Follow-up of each patient/mean/median reported = NA | Mean = NA | HT | AHT | MPR ≥ 80% | RR of stroke |
| Corrao et al., 2017 [3] | Italy;  Nested case-control;  38,461 (60.00) | Start = index date,  End = relevant event or end of follow-up;  Mean= 4 | Mean, elder = 75.0; very elder = 87.8 | HT | AHT | PDC ≥ 75% | OR of stroke, OR of ACM |
| Jackevicius et al., 2017 [11] | Canada; Cohort;  25,976 (52.43) | Start = index date,  End = relevant event or end of follow-up;  Mean, all = NA; dabigatran = 1.46; and rivaroxaban = 0.75 | Mean, all = NA; dabigatran= 80.68 ± 6.70; rivaroxaban= 76.96 ± 7.05 | AF | AT | Adhered continuously without gaps of 14 days or more | HR of stroke/TIA, HR of stroke/TIA or death |
| Fukuda & Mizobe, 2017 [30] | Japan;  Cohort;  11,331 (39.22) | Start = index date,  End = relevant event or end of follow-up;  Mean, all = NA;  adherence = 2.30 ± 1.20; non-adherence = 1.75 ± 0.97 | Mean, all = NA; adherence = 52.3 ± 11.0; non-adherence = 50.0 ± 11.0 | T2DM | AD (including insulin) | Continuously taking medication during “non-adherence observation period”, non-persistence gap ≥ 6 months consecutively | HR of stroke |
| DespHande et al., 2018 [31] | United States; Cohort;  5,575 (30.13) | Refers to outcome follow-up, mean = 0.76 ± 0.57 | Mean = 65 ± 10.5 | AF | AT | PDC ≥ 80% | HR of stroke, HR of bleeding |
| Gatwood et al., 2018 [32] | United States; Cohort;  159,032 (4.40) | Start = index date,  End = relevant event or end of 5-year predetermined follow-up in the study design;  Follow-up of each patient/mean/median reported = NA | Mean, all = NA; non-adherent = 61.7 ± 11.7; adherent = 62.7 ± 10.6 | T2DM (uncomplicated) | AD | PDC ≥ 80% | HR of stroke, HR of ACM |
| Kim et al., 2018 [4] | South Korea; Cohort;  65,067 (38.82) | Start = index date,  End = relevant event or end of 10-year predetermined follow-up in the study design;  Follow-up of each patient/mean/median reported = NA | Range= 40 to > 7 0 | T2DM | AD | PDC ≥ 80% | HR of stroke, HR of ACM |
| Hurtado-Navarro et al., 2018 [33] | Spain;  Cohort;  38,026 (47.38) | Start = index date,  End = relevant event or end of 1-year predetermined follow-up in the study design;  Follow-up of each patient/mean/median reported = NA | Mean = 74.12 ± 9.85 | NVAF | AT | PDC ≥ 80% | HR of stroke, HR of ACM, HR of bleeding |
| McHorney et al., 2019 [34] | United States; Cohort;  54,280 (45.70) | Start = Index date,  End = relevant event or end of 4-year predetermined follow-up in the study design;  Follow-up of each patient/mean/median reported = NA | Mean = 72.6 ± 10.8 | NVAF | AT | PDC ≥ 80% | HR of stroke, HR of bleeding |
| Martinez et al., 2020 [35] | United Kingdom; Nested case-control;  2,626 (48.50) | Start = index date,  End = relevant event or end of 12-year predetermined follow-up in the study design;  Follow-up of each patient/mean/median reported = NA | Mean = 77.4 ± 7.9 | AF | AT | Current medication use /continuously taking medication; Discontinuation > 120 days as of index date | RR of stroke |
| Kim et al., 2020 [36] | South Korea; Cohort;  67,119 (44.80) | Start = index date,  End = relevant event or end of follow-up;  Median (IQR) = 1.05 (0.63–1.4) | Mean = 72.1± 9.4 | NVAF | AT | PDC ≥ 80% | HR of Str, HR of bleeding |
| Hernandez et al., 2020 [37] | United States; Cohort;  39,272 (58.4) | Start = index date,  End = relevant event or end of 0.92-year predetermined follow-up in the study design;  Follow-up of each patient/mean/median reported = NA | Mean = NA | AF (newly diagnosed) | AT | PDC ≥ 80% | RR^*^ of stroke |
| Toorop et al., 2021 [10] | Netherland; Cohort;  93,048 (43.80) | Start = index date,  End = relevant event or end of 1-year predetermined follow-up in the study design;  Follow-up of each patient/mean/median reported = NA | Mean = 72.2 ± 11.1 | NVAF | AT | Non-persistence gap = 100 days | HR of stroke, HR of ACM, HR of Str or death |
| Ryou et al., 2021 [38] | South Korea; Cohort;  11,320 (63.52) | Refers to outcome follow-up, which was predetermined in the study design (i.e., 6.5 years) or until the end of the study;  Follow-up of each patient/mean/median reported = NA | Mean = NA | HL | LLA | Discontinued medication in the first 6 months and did not receive any statin until the end of study | HR of stroke |
| Lee et al., 2021 [39] | South Korea; Cohort;  123,390 (24.87) | Start = index date,  End = relevant event or end of follow-up;  Median = 10 | Mean = NA | HT | AHT | PDC ≥ 80% | HR of stroke |
| Rublee et al., 2012 [2] | United States; Cohort;  79,010 (46.58) | Refers to outcome follow-up, which was predetermined in the study design (i.e., 2 years) or until the end of the study;  Follow-up of each patient/mean/median reported = NA | Mean, all = NA; Adherence = 53.7 ± 10.7; non-adherence = 51.4 ± 11.9 | HL | LLA | PDC ≥ 60% | RR^*^ of stroke |
| Karlsson et al., 2018 [40] | Swedish; Cohort;  74,909 (34.20) | Start = index date,  End = relevant event or end of follow-up;  Mean = 3.6 | Mean = 68.5 ± 10.2 | T2DM | LLA | MPR > 80% | HR of stroke, HR of ACM |

^*^ The risk was calculated from the event and sample size which were available from studies without any adjustment; the calculation formula followed Cochrane Handbook for risk ratio and odd ratio calculation [41]. Abbreviations: ACM = all-cause mortality; AD = anti-diabetic; AHT = anti-hypertension; AT = anti-thrombotic; CMA = cumulative medication adherence; HR = hazard ratio; HL = hyperlipidemia; HS = haemorrhagic stroke; IQR = interquartile range; IS = ischemic stroke; LLA = lipid lowering agent; MMAS = the Morisky Medication Adherence Scale; MPR = medication possession ratio; MRA = medication refill adherence; NA = not available; NVAF= non-valvular atrial fibrillation; OR = odd ratio; PDC = proportion of days covered; RR = relative risk; T2DM = type 2 diabetes mellitus; TIA = transient ischemic attack

# **Table S6a**. Relative risk of medication non-adherence impact on stroke-associated outcomes stratified by the group of medications used for major modifiable stroke-related diseases or thrombosis prevention

| **Clinical Outcomes** | **Category of medications** | **number of studies** | **number of participants** | **Relative risk**  **(95% CI)** | **Heterogeneity** | | **Specific name/class of medications** |
| --- | --- | --- | --- | --- | --- | --- | --- |
|  |  |  |  |  | **I^2^ (%)** | **p-value** |  |
| Stroke occurrence | Antidiabetic [4, 32] | 2 | 224,099 | 1.312  (1.257, 1.369) | 0.00 | 0.326 | Biguanide, sulfonylurea, and others. (1 study not informed) |
|  | Antidiabetic & not specify diabetes medication [30] | 1 | 11,331 | 1.260  (0.881, 1.802) | 0.00 | 1.000 | Not diabetes medication, non-insulin diabetes medication, and insulin.  Non-insulin diabetes medications are referring to sulfonylureas, biguanides, thiazolidinedione, alpha-glucosidase inhibitors, glinides, dipeptidyl peptidase-4 inhibitors, and glucagon-like peptide-1 agonists. |
|  | Antihypertensive [1, 3, 5-7, 9, 12, 13, 15, 19, 20, 27, 29, 39] | 14 | 752,793 | 1.421  (1.166, 1.732) | 99.01 | < 0.001 | Diuretics, beta-blockers, CCBs, ARBs, ACE inhibitors, vasodilators, alpha blockers, miscellaneous antihypertension agents, and combination. (9 studies not informed) |
|  | Antithrombotic [10, 11, 17, 21, 24, 26, 31, 33-37] | 12 | 468,600 | 1.852  (1.583, 2.166) | 89.28 | < 0.001 | Warfarin, dabigatran, rivaroxaban, or apixaban, acenocoumarol. (2 studies not informed) |
|  | Lipid-lowering agents [2, 14, 22, 23, 38, 40] | 6 | 383,408 | 1.318  (1.017, 1.708) | 98.99 | < 0.001 | Statin. (1 study not informed) |
|  | Lipid-lowering agents & or Antihypertensive [25] | 1 | 58,266 | 2.040  (1.716, 2.425) | 0.00 | 1.000 | Statin and/or AHT (not specify). |
| Either stroke or death | Antithrombotic [11, 28] | 2 | 28,858 | 1.750  (1.564, 1.957) | 22.46 | 0.256 | Dabigatran, rivaroxaban. |
|  | Not available information of group medication [16] | 1 | 1,114 | 2.176  (1.147, 4.129) | 0.00 | 1.000 | Not specify. |
| All-cause mortality | Antidiabetic [4, 32] | 2 | 224,099 | 1.281  (1.151, 1.426) | 90.15 | 0.001 | Biguanide, sulfonylurea, and other (not specify). (1 study not informed) |
|  | Antihypertensive [1, 3, 6, 7, 18, 20] | 6 | 411,429 | 1.421  (1.209, 1.671) | 97.61 | < 0.001 | CCB, beta-blockers, diuretics, ACEIs, ARBs, and a combination. (2 studies not informed) |
|  | Antithrombotic [10, 33] | 2 | 131,074 | 1.686  (0.898, 3.166) | 98.75 | < 0.001 | OAC (all types DOAC or VKA therapy). |
|  | Lipid-lowering agents [22, 40] | 2 | 172484 | 1.234  (0.970, 1.570) | 91.64 | 0.001 | Statin. (1 study not informed) |

AHT: antihypertensive; ACEI: angiotensin converting enzyme inhibitor; ARB: angiotensin II receptor blockers; CCB: calcium channel blocker; DOAC: direct oral anticoagulant; NR: not eported; OAC: oral anticoagulant; VKA: vitamin-K antagonist.

# **Table S6b.** Relative risk of medication non-adherence impact on stroke-associated outcomes without stratification of medication used for major modifiable diseases or thrombosis prevention

| **Clinical Outcomes** | **Category of medication** | **number of studies** | **number of participants** | **Relative risk (95% CI)** | **p-value** | **I^2^** | **Specific name/class of medications** | **Rationale** |
| --- | --- | --- | --- | --- | --- | --- | --- | --- |
| Bleeding | Antithrombotic[17, 24, 31, 33, 34, 36] | 6 | 247,359 | 0.894  (0.803, 0.996) | 0.043 | 64.31 | Warfarin, acenocoumarol, dabigatran, rivaroxaban, or apixaban | Similar group medication assessed from corresponding studies |

# **Table S7.** Subgroup analysis of medication non-adherence impact associated with major modifiable diseases on stroke events and all-cause mortality risks

| **Risk\Subgroup** | **number of studies** | **Relative risk (RR) (95% CI)** | **P-value within subgroup** | **I^2^ (%)** | **p-value between subgroup** | | **Interpretation of p-value between subgroup/additional information** |
| --- | --- | --- | --- | --- | --- | --- | --- |
| **Stroke occurrence** |  |  |  |  |  |  | |
| Age distribution, years old  < 65  ≥ 65 | 13  17 | 1.408 (1.153, 1.718) 1.682 (1.413, 2.003) | < 0.001  < 0.001 | 98.44  98.47 | 0.188 | Impact of non-adherence associated with disease risk factor on occurrence of stroke was not varied by age | |
| Sample size  < 50,000  ≥ 50,000 | 18  18 | 1.499 (1.305, 1.722) 1.554 (1.364, 1.770) | < 0.001  < 0.001 | 94.73 98.56 | 0.713 | Impact of non-adherence associated with disease risk factor on occurrence of stroke was not varied by sample size | |
| Follow-up period (years)  < 2  ≥ 2 | 7  22 | 1.711 (1.352, 2.166) 1.406 (1.236, 1.599) | < 0.001  < 0.001 | 96.91  98.67 | 0.152 | Impact of non-adherence associated with disease risk factor on occurrence of stroke was not varied by follow-up period | |
| Proportion of female (%)  < 50  ≥ 50 | 20  16 | 1.519 (1.314, 1.755) 1.548 (1.317, 1.821) | < 0.001  < 0.001 | 97.05 98.89 | 0.862 | Impact of non-adherence associated with disease risk factor on occurrence of stroke was not varied by proportion of female | |
| Quality of study  < 8  ≥ 8 | 9  27 | 1.539 (1.253, 1.891) 1.528 (1.353, 1.727) | < 0.001  < 0.001 | 99.07  97.31 | 0.954 | Impact of non-adherence associated with disease risk factor on occurrence of stroke was not varied by quality of study | |
| **All-cause mortality** |  |  |  |  |  |  | |
| Age distribution, years old  < 65  ≥ 65 | 6  3 | 1.312 (1.156, 1.489)  1.602 (1.341, 1.915) | < 0.001  < 0.001 | 95.66  98.78 | 0.073 | Removed from sensitivity analysis due to total studies which can compare <10 | |
| Sample size  < 50,000  ≥ 50,000 | 6  6 | 1.405 (1.161, 1.701)  1.406 (1.164, 1.699) | < 0.001  < 0.001 | 97.53  99.15 | 0.996 | Impact of non-adherence associated with disease risk factor on all-cause mortality was not varied by sample size | |
| Follow-up period (years)  < 2  ≥ 2 | 2  8 | 1.497 (1.228, 1.824)  1.305 (1.189, 1.433) | < 0.001  < 0.001 | 95.30 97.36 | 0.220 | Removed from sensitivity analysis due to unbalanced subgroup | |
| Proportion of female (%)  < 50  ≥ 50 | 6  6 | 1.400 (1.157, 1.694)  1.412 (1.166, 1.709) | 0.001  < 0.001 | 99.14  97.56 | 0.952 | Impact of non-adherence associated with disease risk factor on all-cause mortality was not varied by proportion of female | |
| Quality of study  < 8  ≥ 8 | 1  11 | 1.410 (0.945, 2.103)  1.405 (1.243, 1.587) | 0.092  < 0.001 | 0.0  98.68 | 0.986 | Removed from sensitivity analysis due to unbalanced subgroup. | |

**Table S8a.** Sensitivity analysis of stroke-associated outcomes as a consequence of medication non-adherence when one study was removed at one time

| **Study reporting clinical outcomes associated with stroke** | **Relative risk (95% CI) result if one study removed** | **p-value** |
| --- | --- | --- |
| 1. **Occurrence of stroke** |  |  |
| Rublee et al., 2012 | 1.551 (1.397, 1.722) | < 0.001 |
| Cumming et al., 2013 | 1.548 (1.402, 1.709) | < 0.001 |
| Corrao et al., 2017 | 1.547 (1.394, 1.717) | < 0.001 |
| Bailey et al., 2010 | 1.545 (1.394, 1.714) | < 0.001 |
| Dragomir et al., 2010 | 1.545 (1.392, 1.714) | < 0.001 |
| Korhonen et al., 2016 | 1.543 (1.390, 1.712) | < 0.001 |
| Lee et al., 2017 | 1.543 (1.389, 1.715) | < 0.001 |
| Esposti et al., 2011 | 1.542 (1.391, 1.710) | < 0.001 |
| Perreault et al., 2009 | 1.541 (1.389, 1.710) | < 0.001 |
| Gatwood et al., 2018 | 1.541 (1.389, 1.709) | < 0.001 |
| Kim et al., 2016 | 1.540 (1.387, 1.709) | < 0.001 |
| Kettani et al., 2009 | 1.540 (1.387, 1.709) | < 0.001 |
| Breekveldt-Postma et al., 2008 | 1.539 (1.387, 1.707) | < 0.001 |
| Kim et al., 2018 | 1.539 (1.381, 1.714) | < 0.001 |
| Fukuda & Mizobe, 2017 | 1.537 (1.387, 1.704) | < 0.001 |
| Kim et al., 2020 | 1.536 (1.383, 1.706) | < 0.001 |
| Rannanheimo et al., 2015 | 1.532 (1.381, 1.699) | < 0.001 |
| Hernandez et al., 2020 | 1.532 (1.381, 1.699) | < 0.001 |
| McHorney et al., 2019 | 1.531 (1.381, 1.699) | < 0.001 |
| Shin et al., 2013 | 1.531 (1.380, 1.698) | < 0.001 |
| Alberts et al., 2016 | 1.531 (1.380, 1.698) | < 0.001 |
| Hurtado-Navarro et al., 2018 | 1.530 (1.381, 1.696) | < 0.001 |
| Spivey et al., 2015 | 1.530 (1.379, 1.696) | < 0.001 |
| Ryou et al., 2021 | 1.528 (1.379, 1.693) | < 0.001 |
| Karlsson et al., 2018 | 1.526 (1.376, 1.693) | < 0.001 |
| Fitch et al., 2012 | 1.525 (1.376, 1.691) | < 0.001 |
| Lee et al., 2021 | 1.525 (1.375, 1.691) | < 0.001 |
| Liu et al., 2009 | 1.524 (1.375, 1.690) | < 0.001 |
| Desphande et al., 2018 | 1.524 (1.375, 1.689) | < 0.001 |
| Toorop et al., 2021 | 1.523 (1.374, 1.689) | < 0.001 |
| Herttua et al., 2016 | 1.517 (1.369, 1.682) | < 0.001 |
| Martinez et al., 2020 | 1.509 (1.362, 1.672) | < 0.001 |
| Yao et al., 2016 | 1.501 (1.355, 1.662) | < 0.001 |
| Herttua et al., 2013 | 1.500 (1.365, 1.647) | < 0.001 |
| Yang et al., 2016 | 1.498 (1.359, 1.650) | < 0.001 |
| Jackevicius et al., 2017 | 1.492 (1.348, 1.652) | < 0.001 |
| **Pooled relative risk (Basic finding)** | 1.530 (1.382, 1.694) | < 0.001 |
| **Conclusion of sensitivity analysis:** | Consistent with the basic pooled analysis | |
| 1. **Either stroke occurrence or death** | | |
| Jackevicius et al., 2017 | 1.620 (1.297, 2.024) | < 0.001 |
| Park & Sohn, 2011 | 1.750 (1.564, 1.957) | < 0.001 |
| Borne et al., 2017 | 1.804 (1.666, 1.954) | < 0.001 |
| **Pooled relative risk (Basic finding)** | 1.777 (1.648, 1.917) | < 0.001 |
| **Conclusion of sensitivity analysis:** | Consistent with the basic pooled analysis | |
| 1. **Bleeding** |  |  |
| Yao et al., 2016 | 0.933 (0.867, 1.005) | 0.067 |
| Fitch et al., 2012 | 0.911 (0.796, 1.042) | 0.172 |
| Hurtado-Navarro et al., 2018 | 0.891 (0.794, 1.001) | 0.052 |
| McHorney et al., 2019 | 0.891 (0.775, 1.025) | 0.105 |
| DespHande et al., 2018 | 0.877 (0.782, 0.983) | 0.024 |
| Kim et al., 2020 | 0.866 (0.772, 0.972) | 0.014 |
| **Pooled relative risk (Basic finding)** | 0.894 (0.803, 0.996) | 0.043 |
| **Conclusion of sensitivity analysis:** | Consistent with the basic pooled analysis | |
| 1. **All-cause mortality** |  |  |
| Toorop et al., 2021 | 1.337 (1.231, 1.452) | < 0.001 |
| Esposti et al., 2011 | 1.374 (1.222, 1.544) | < 0.001 |
| Kim et al., 2016 | 1.392 (1.236, 1.568) | < 0.001 |
| Shin et al., 2013 | 1.399 (1.241, 1.577) | < 0.001 |
| Corrao et al., 2017 | 1.405 (1.243, 1.587) | < 0.001 |
| Rannanheimo et al., 2015 | 1.405 (1.246, 1.584) | < 0.001 |
| Kim et al., 2018 | 1.411 (1.242, 1.603) | < 0.001 |
| Wong et al., 2013 | 1.413 (1.250, 1.599) | < 0.001 |
| Hurtado-Navarro et al., 2018 | 1.422 (1.261, 1.604) | < 0.001 |
| Gatwood et al., 2018 | 1.425 (1.258, 1.614) | < 0.001 |
| Karlsson et al., 2018 | 1.438 (1.255, 1.649) | < 0.001 |
| Bailey et al., 2010 | 1.441 (1.257, 1.652) | < 0.001 |
| **Pooled relative risk (Basic finding)** | 1.405 (1.253, 1.576) | < 0.001 |
| **Conclusion of sensitivity analysis:** | Consistent with the basic pooled analysis | |

**Table S8b.** Sensitivity analysis of stroke occurrence as a consequence of medication non-adherence after removing 6 unadjusted risks from the pooled analysis

| **Study reporting adjusted risks** | **Adjusted relative risk (95% CI)** | **p-value** |
| --- | --- | --- |
| Cumming et al., 2013 | 1.030 (1.010, 1.050) | 0.003 |
| Corrao et al., 2017 | 1.080 (0.998, 1.168) | 0.055 |
| Bailey et al., 2010 | 1.090 (0.930, 1.278) | 0.287 |
| Dragomir et al., 2010 | 1.130 (1.026, 1.245) | 0.013 |
| Esposti et al., 2011 | 1.170 (0.982, 1.393) | 0.078 |
| Lee et al., 2017 | 1.180 (1.114, 1.250) | < 0.001 |
| Gatwood et al., 2018 | 1.220 (1.049, 1.419) | 0.010 |
| Kim et al., 2016 | 1.250 (1.141, 1.370) | < 0.001 |
| Fukuda & Mizobe, 2017 | 1.260 (0.881, 1.802) | 0.206 |
| Breekveldt-Postma et al., 2008 | 1.280 (1.130, 1.450) | < 0.001 |
| Kim et al., 2018 | 1.320 (1.263, 1.380) | < 0.001 |
| Kim et al., 2020 | 1.370 (1.278, 1.468) | < 0.001 |
| McHorney et al., 2019 | 1.490 (1.291, 1.720) | < 0.001 |
| Rannanheimo et al., 2015 | 1.490 (1.325, 1.676) | < 0.001 |
| Alberts et al., 2016 | 1.500 (1.300, 1.730) | < 0.001 |
| Shin et al., 2013 | 1.510 (1.285, 1.774) | < 0.001 |
| Hurtado-Navarro et al., 2018 | 1.520 (0.986, 2.344) | 0.058 |
| Spivey et al., 2015 | 1.550 (1.329, 1.808) | < 0.001 |
| Karlsson et al., 2018 | 1.670 (1.630, 1.710) | < 0.001 |
| Ryou et al.,2021 | 1.700 (0.834, 3.466) | 0.144 |
| Fitch et al.,2012 | 1.710 (1.398, 2.092) | < 0.001 |
| Lee et al., 2021 | 1.720 (1.549, 1.909) | < 0.001 |
| Liu et al., 2009 | 1.740 (1.547, 1.957) | < 0.001 |
| Toorop et al., 2021 | 1.790 (1.490, 2.150) | < 0.001 |
| Desphande et al., 2018 | 1.820 (1.240, 2.671) | 0.002 |
| Herttua et al., 2016 | 2.040 (1.716, 2.425) | < 0.001 |
| Martinez et al., 2020 | 2.600 (1.992, 3.393) | < 0.001 |
| Herttua et al., 2013 | 2.700 (2.526, 2.886) | < 0.001 |
| Yao et al., 2016 | 3.140 (2.456, 4.014) | < 0.001 |
| Jackevicius et al., 2017 | 4.290 (3.125, 5.890) | < 0.001 |
| **Pooled relative risk** | 1.558 (1.395, 1.740) | < 0.001 |
| **Conclusion of sensitivity analysis:** | consistent with the basic pooled analysis | |

**Fig. S1** Pooled risk estimates of the safety outcome associated with non-adherence versus adherence to medications among patients taking medications for primary stroke prevention without disease or medication stratification


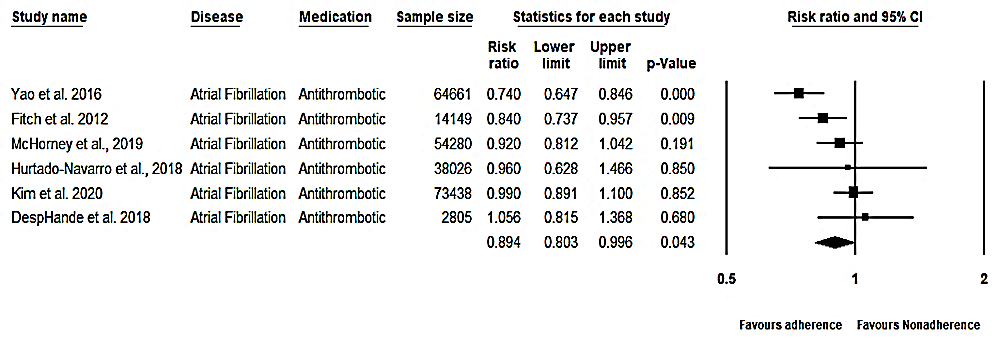
 The pooled risk estimate was presented as relative risk. Overall heterogeneity (I^2^) = 64.31%. Six studies [17, 24, 31, 33, 34, 36] investigated similar diseases and medications, i.e., atrial fibrillation and antithrombotic agents (particularly oral anticoagulants). Yao et al., 2016 reported warfarin, dabigatran, rivaroxaban, or apixaban. Fitch et al., 2012 reported warfarin only. Hurtado-Navarro et al., 2018 reported acenocoumarol, apixaban, dabigatran or rivaroxaban. McHorney et al., 2019, Kim et al., 2020 and DespHande et al., 2018 reported NOACs.

**Fig. S2** Funnel plot of the relative risk of the impact of medication non-adherence associated major modifiable stroke-related diseases on stroke occurrence


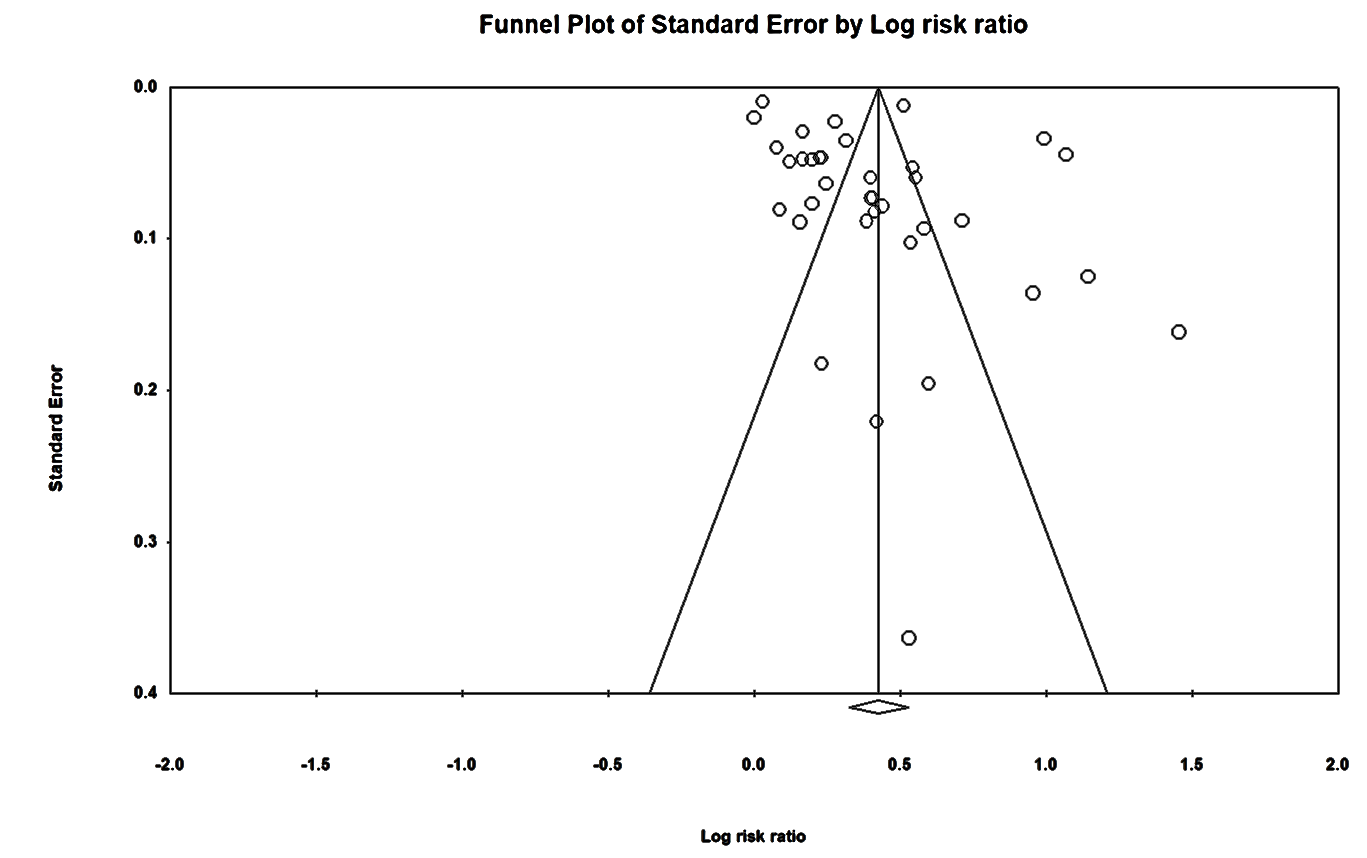


The funnel plot presents asymmetry. The point estimate relative risk was 1.530 (95% CI: 1.382, 1.694), and the Egger’s regression intercept was 3.700 (p-value = 0.055).

**Fig. S3** Funnel plot of the relative risk of the impact of medication non-adherence associated with major modifiable stroke-related diseases on all-cause mortality


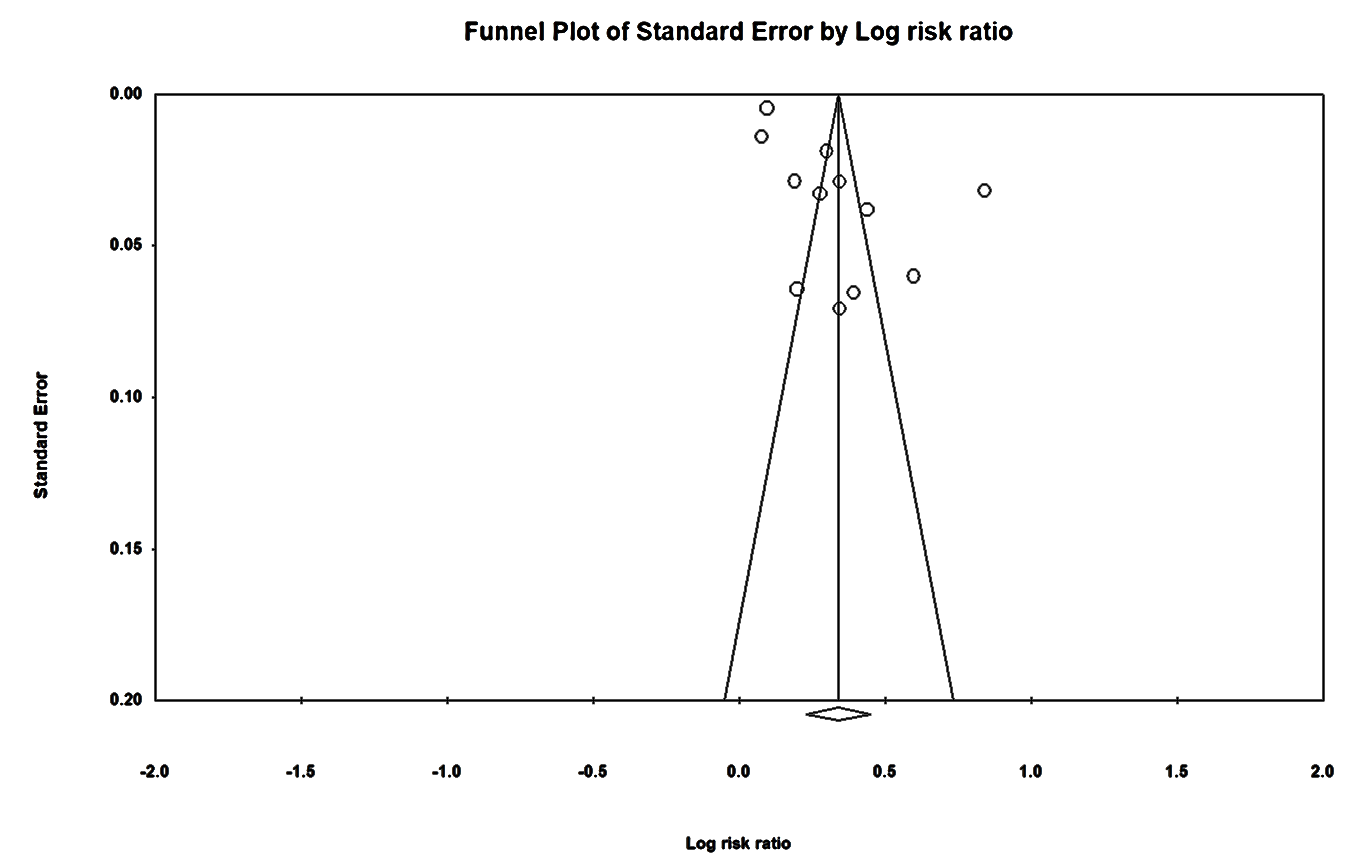
The funnel plot presents asymmetry. The point estimate of the relative risk was 1.405 (95% CI: 1.253-1.576), and the Egger’s regression intercept was 9.098 (p-value = 0.008).

**References of the supplementary materials**

1. Degli Esposti L, Saragoni S, Benemei S, Batacchi P, Geppetti P, Di Bari M, et al (2011) **Adherence to antihypertensive medications and health outcomes among newly treated hypertensive patients.** *Clinicoecon Outcomes Res* **3**:47-54. <https://doi.org/10.2147/ceor.s15619>
2. Rublee DA, Chen SY, Mardekian J, Wu N, Rao P, Boulanger L (2012) **Evaluation of cardiovascular morbidity associated with adherence to atorvastatin therapy.** *Am J Ther*. **19**(1):24-32. <https://doi.org/10.1097/MJT.0b013e3181ee707e>
3. Corrao G, Rea F, Monzio Compagnoni M, Merlino L, Mancia G (2017) **Protective effects of antihypertensive treatment in patients aged 85 years or older.** J *Hypertens* **35**(7):1432-1441. <https://doi.org/10.1097/hjh.0000000000001323>
4. Kim YY, Lee JS, Kang HJ, Park SM (2018) **Effect of medication adherence on long-term all-cause-mortality and hospitalization for cardiovascular disease in 65,067 newly diagnosed type 2 diabetes patients**. *Sci Rep* **8**(1):12190. <https://doi.org/10.1038/s41598-018-30740-y>
5. Kettani FZ, Dragomir A, Côté R, Roy L, Bérard A, Blais L, et al (2009) **Impact of a better adherence to antihypertensive agents on cerebrovascular disease for primary prevention.** *Stroke* **40**(1):213-220. <https://doi.org/10.1161/strokeaha.108.522193>
6. Bailey JE, Wan JY, Tang J, Ghani MA, Cushman WC (2010) **Antihypertensive medication adherence, ambulatory visits, and risk of stroke and death**. *J Gen Intern Med* **25**(6):495-503. <https://doi.org/10.1007/s11606-009-1240-1>
7. Kim S, Shin DW, Yun JM, Hwang Y, Park SK, Ko YJ, et al (2016) **Medication adherence and the risk of cardiovascular mortality and hospitalization among patients with newly prescribed antihypertensive medications**. *Hypertension* **67**(3):506-512. <https://doi.org/10.1161/hypertensionaha.115.06731>
8. Steiner JF, Prochazka AV (1997) **The assessment of refill compliance using pharmacy records: methods, validity, and applications.** *J Clin Epidemiol* **50**(1):105-116. <https://doi.org/10.1016/S0895-4356(96)00268-5>
9. Liu PH, Hu FC, Wang JD (2009) **Differential risks of stroke in pharmacotherapy on uncomplicated hypertensive patients?** *J Hypertens* **27**(1):174-180. <https://doi.org/10.1097/hjh.0b013e3283193a29>
10. Toorop MMA, Chen Q, Tichelaar VYIG, Cannegieter SC, Lijfering WM (2021) **Predictors, time course, and outcomes of persistence patterns in oral anticoagulation for non-valvular atrial fibrillation: a Dutch Nationwide Cohort Study.** *Eur Heart J* **42**(40):4126-4137. <https://doi.org/10.1093/eurheartj/ehab421>
11. Jackevicius CA, Tsadok MA, Essebag V, Atzema C, Eisenberg MJ, Tu JV, et al (2017) **Early non-persistence with dabigatran and rivaroxaban in patients with atrial fibrillation**. *Heart* **103**(17):1331-1338. <https://doi.org/10.1136/heartjnl-2016-310672>
12. Cummings DM, Letter AJ, Howard G, Howard VJ, Safford MM, Prince V, et al (2013) **Medication adherence and stroke/TIA risk in treated hypertensives: results from the REGARDS study.** *J Am Soc Hypertens* **7**(5):363-369. <https://doi.org/10.1016/j.jash.2013.05.002>
13. Breekveldt-Postma NS, Penning-van Beest FJ, Siiskonen SJ, Falvey H, Vincze G, Klungel OH, et al (2008) **The effect of discontinuation of antihypertensives on the risk of acute myocardial infarction and stroke**. *Curr Med Res Opin* **24**(1):121-127. <https://doi.org/10.1185/030079908x253843>
14. Perreault S, Ellia L, Dragomir A, Côté R, Blais L, Bérard A, et al (2009) **Effect of statin adherence on cerebrovascular disease in primary prevention.** *Am J Med* **122**(7):647-655. <https://doi.org/10.1016/j.amjmed.2009.01.032>
15. Dragomir A, Côté R, Roy L, Blais L, Lalonde L, Bérard A, et al (2010) **Impact of adherence to antihypertensive agents on clinical outcomes and hospitalization costs**. *Med Care* **48**(5):418-425. <https://doi.org/10.1097/MLR.0b013e3181d567bd>
16. Park IS, Sohn HS (2011) **Effect of medicine adherence on the occurrence of cerebrovascular disorders in diabetes mellitus patients**. *Epidemiol Health* **33**:e2011001. <https://doi.org/10.4178/epih/e2011001>
17. Fitch K, Broulette J, Pyenson B, Iwasaki K, Kwong WJ (2012) **Utilization of anticoagulation therapy in medicare patients with nonvalvular atrial fibrillation.** *Am Health Drug Benefits* **5**(3):157-168.
18. Wong MC, Tam WW, Cheung CS, Wang HH, Tong EL, Sek AC, et al (2013) **Drug adherence and the incidence of coronary heart disease- and stroke-specific mortality among 218,047 patients newly prescribed an antihypertensive medication: a five-year cohort study.** *Int J Cardiol* **168**(2):928-933. <https://doi.org/10.1016/j.ijcard.2012.10.048>
19. Herttua K, Tabák AG, Martikainen P, Vahtera J, Kivimäki M (2013) **Adherence to antihypertensive therapy prior to the first presentation of stroke in hypertensive adults: population-based study.** *Eur Heart J* **34**(38):2933-2939. <https://doi.org/10.1093/eurheartj/eht219>
20. Shin S, Song H, Oh S-K, Choi KE, Kim H, Jang S (2013) **Effect of antihypertensive medication adherence on hospitalization for cardiovascular disease and mortality in hypertensive patients.** *Hypertens Res* **36**(11):1000-1005. <https://doi.org/10.1038/hr.2013.85>
21. Spivey CA, Liu X, Qiao Y, Mardekian J, Parker RB, Phatak H, et al (2015) **Stroke associated with discontinuation of warfarin therapy for atrial fibrillation**. *Curr Med Res Opin* **31**(11):2021-2029. <https://doi.org/10.1185/03007995.2015.1082995>
22. Rannanheimo PK, Tiittanen P, Hartikainen J, Helin-Salmivaara A, Huupponen R, Vahtera J, et al (2015) **Impact of statin adherence on cardiovascular morbidity and all-cause mortality in the primary prevention of cardiovascular disease: a population-based cohort study in Finland.** *Value Health* **18**(6):896-905. <https://doi.org/10.1016/j.jval.2015.06.002>
23. Korhonen MJ, Ruokoniemi P, Ilomäki J, Meretoja A, Helin-Salmivaara A, Huupponen R (2016) **Adherence to statin therapy and the incidence of ischemic stroke in patients with diabetes.** *Pharmacoepidemiol Drug Saf* **25**(2):161-169. <https://doi.org/10.1002/pds.3936>
24. Yao X, Abraham NS, Alexander GC, Crown W, Montori VM, Sangaralingham LR, et al (2016) **Effect of adherence to oral anticoagulants on risk of stroke and major bleeding among patients with atrial fibrillation**. *J Am Heart Assoc* **5**(2):e003074. <https://doi.org/10.1161/jaha.115.003074>
25. Herttua K, Martikainen P, Batty GD, Kivimäki M (2016) **Poor adherence to statin and antihypertensive therapies as isk factors for fatal stroke**. *J Am Coll Cardiol* **67**(13):1507-1515. <https://doi.org/10.1016/j.jacc.2016.01.044>
26. Alberts MJ, Peacock WF, Fields LE, Bunz TJ, Nguyen E, Milentijevic D, et al (2016) **Association between once- and twice-daily direct oral anticoagulant adherence in nonvalvular atrial fibrillation patients and rates of ischemic stroke**. *Int J Cardiol* **215**:11-13. <https://doi.org/10.1016/j.ijcard.2016.03.212>
27. Yang Z, Howard DH, Will J, Loustalot F, Ritchey M, Roy K (2016) **Association of antihypertensive medication adherence with healthcare use and Medicaid expenditures for acute cardiovascular events.** *Med Car* **54**(5):504-511. <https://doi.org/10.1097/mlr.0000000000000515>
28. Borne RT, O’Donnell C, Turakhia MP, Varosy PD, Jackevicius CA, Marzec LN, et al (2017) **Adherence and outcomes to direct oral anticoagulants among patients with atrial fibrillation: findings from the veterans health administration**. *BMC Cardiovasc Disor* **17**(1):236. <https://doi.org/10.1186/s12872-017-0671-6>
29. Lee HJ, Jang SI, Park EC (2017) **Effect of adherence to antihypertensive medication on stroke incidence in patients with hypertension: a population-based retrospective cohort study.** *BMJ Open* **7**(6):e014486. <https://doi.org/10.1136/bmjopen-2016-014486>
30. Fukuda H, Mizobe M (2017) **Impact of nonadherence on complication risks and healthcare costs in patients newly-diagnosed with diabetes**. *Diabetes Res Clin Pract* **123**:55-62. <https://doi.org/10.1016/j.diabres.2016.11.007>
31. Deshpande CG, Kogut S, Laforge R, Willey C (2018) **Impact of medication adherence on risk of ischemic stroke, major bleeding and deep vein thrombosis in atrial fibrillation patients using novel oral anticoagulants.** *Curr Med Res Opin* **34**(7):1285-1292. <https://doi.org/10.1080/03007995.2018.1428543>
32. Gatwood JD, Chisholm-Burns M, Davis R, Thomas F, Potukuchi P, Hung A, et al (2018) **Differences in health outcomes associated with initial adherence to oral antidiabetes medications among veterans with uncomplicated type 2 diabetes: a 5-year survival analysis.** *Diabet Med* **35**(11):1571-1579. <https://doi.org/10.1111/dme.13775>
33. Hurtado-Navarro I, García-Sempere A, Rodríguez-Bernal C, Santa-Ana-Tellez Y, Peiró S, Sanfélix-Gimeno G (2018) **Estimating adherence based on prescription or dispensation information: impact on thresholds and outcomes.** *Front Pharma*col **9**:1353. <https://doi.org/10.3389/fphar.2018.01353>
34. McHorney CA, Peterson ED, Ashton V, Laliberté F, Crivera C, Germain G, et al (2019) **Modeling the impact of real-world adherence to once-daily (QD) versus twice-daily (BID) non-vitamin K antagonist oral anticoagulants on stroke and major bleeding events among non-valvular atrial fibrillation patients.** *Curr Med Res Opin* **35**(4):653-660. <https://doi.org/10.1080/03007995.2018.1530205>
35. Martinez C, Wallenhorst C, Rietbrock S, Freedman B (2020) **Ischemic stroke and transient ischemic attack risk following vitamin K antagonist cessation in newly diagnosed atrial fibrillation: a cohort study.** *J Am Heart Assoc* **9**(2):e014376. <https://doi.org/10.1161/JAHA.119.014376>
36. Kim D, Yang PS, Jang E, Yu HT, Kim TH, Uhm JS, et al (2020) **The optimal drug adherence to maximize the efficacy and safety of non-vitamin K antagonist oral anticoagulant in real-world atrial fibrillation patients.** *Europace* **22**(4):547-557. <https://doi.org/10.1093/europace/euz273>
37. Hernandez I, He M, Brooks MM, Saba S, Gellad WF, et al (2020) **Adherence to anticoagulation and risk of stroke among Medicare beneficiaries newly diagnosed with atrial fibrillation**. *Am J Cardiovasc Drugs* **20**(2):199-207. <https://doi.org/10.1007/s40256-019-00371-3>
38. Ryou IS, Chang J, Son JS, Ko A, Choi S, Kim K, et al (2021) **Association between CVDs and initiation and adherence to statin treatment in patients with newly diagnosed hypercholesterolaemia: a retrospective cohort study**. *BMJ Open* **11**(4):e045375. <https://doi.org/10.1136/bmjopen-2020-045375>
39. Lee H, Yano Y, Cho SMJ, Heo JE, Kim D-W, Park S, et al (2021) **Adherence to antihypertensive medication and incident cardiovascular events in young adults with hypertension**. *Hypertension* **77**(4):1341-1349. <https://doi.org/10.1161/HYPERTENSIONAHA.120.16784>
40. Karlsson SA, Hero C, Svensson AM, Franzén S, Miftaraj M, Gudbjörnsdottir S, et al (2018) **Association between refill adherence to lipid-lowering medications and the risk of cardiovascular disease and mortality in Swedish patients with type 2 diabetes mellitus: a nationwide cohort study.** *BMJ Open* **8**(3):e020309. <https://doi.org/10.1136/bmjopen-2017-020309>
41. Higgins JP, Tianjing Li, Jonathan J Deeks (2022) **Chapter 6: Choosing effect measures and computing estimates of effect.** In: Higgins JPT, Thomas J, Chandler J, Cumpston M, Li T, Page MJ, Welch VA (editors). Cochrane Handbook for Systematic Reviews of Interventions Available from: [www.training.cochrane.org/handbook. Cited on Nov 1](http://www.training.cochrane.org/handbook.%20Cited%20on%20Nov%201), 2022.
